# An Adaptive Generalized Leaky Integrate-and-Fire Model for Hippocampal CA1 Pyramidal Neurons and Interneurons

**DOI:** 10.1007/s11538-023-01206-8

**Published:** 2023-10-04

**Authors:** Addolorata Marasco, Emiliano Spera, Vittorio De Falco, Annalisa Iuorio, Carmen Alina Lupascu, Sergio Solinas, Michele Migliore

**Affiliations:** 1 Department of Mathematics and Applications, University of Naples Federico II, Via Cintia ed. 5A, 80126 Naples, Italy; 2 Institute of Biophysics, National Research Council, Via Ugo La Malfa 153, 90146 Palermo, Italy; 3 Scuola Superiore Meridionale, Largo San Marcellino 10, 80138 Naples, Napoli Italy; 4 Istituto Nazionale di Fisica Nucleare di Napoli, Via Cintia ed. 6, 80126 Naples, Napoli Italy; 5 Faculty of Mathematics, University of Vienna, Oskar-Morgenstern-Platz 1, 1090 Vienna, Austria; 6 Department of Biomedical Science, University of Sassari, Viale San Pietro 23, 07100 Sassari, Italy; 7 Department of Engineering, Parthenope University of Naples, Centro Direzionale - Isola C4, 80143 Naples, Italy

**Keywords:** Neuronal modeling, Generalized leaky integrate-and-fire models, Hippocampus, CA1 pyramidal neurons and interneurons, Constant and piecewise constant stimulations, Neuron firing properties

## Abstract

Full-scale morphologically and biophysically realistic model networks, aiming at modeling multiple brain areas, provide an invaluable tool to make significant scientific advances from in-silico experiments on cognitive functions to digital twin implementations. Due to the current technical limitations of supercomputer systems in terms of computational power and memory requirements, these networks must be implemented using (at least) simplified neurons. A class of models which achieve a reasonable compromise between accuracy and computational efficiency is given by generalized leaky integrate-and fire models complemented by suitable initial and update conditions. However, we found that these models cannot reproduce the complex and highly variable firing dynamics exhibited by neurons in several brain regions, such as the hippocampus. In this work, we propose an adaptive generalized leaky integrate-and-fire model for hippocampal CA1 neurons and interneurons, in which the nonlinear nature of the firing dynamics is successfully reproduced by linear ordinary differential equations equipped with nonlinear and more realistic initial and update conditions after each spike event, which strictly depends on the external stimulation current. A mathematical analysis of the equilibria stability as well as the monotonicity properties of the analytical solution for the membrane potential allowed (i) to determine general constraints on model parameters, reducing the computational cost of an optimization procedure based on spike times in response to a set of constant currents injections; (ii) to identify additional constraints to quantitatively reproduce and predict the experimental traces from 85 neurons and interneurons in response to any stimulation protocol using constant and piecewise constant current injections. Finally, this approach allows to easily implement a procedure to create infinite copies of neurons with mathematically controlled firing properties, statistically indistinguishable from experiments, to better reproduce the full range and variability of the firing scenarios observed in a real network.

**Supplementary Information:**

The online version contains supplementary material available at 10.1007/s11538-023-01206-8.

## Introduction

Hippocampal CA1 pyramidal neurons and interneurons exhibit complex and highly variable firing dynamics (such as adapting, non-adapting, and bursting) that play a key role in modulating the dynamics of the network to which they belong. These patterns can be successfully reproduced by morphologically and biophysically realistic neuron models based on the Hodgkin-Huxley equations (Golomb et al 2006; Bianchi et al 2012; Migliore et al 2018); however, due to the large amount of (nonlinear) equations involved, their computational cost is fairly high. This is an important issue, because large full-scale networks, aiming at modeling multiple brain areas, must be implemented using (at least) simplified neurons, given the actual technical limitations of supercomputing systems.

An alternative modelling option, to reach a balance between accuracy and efficiency, is represented by point-neuron models. In this framework, nonlinear adaptive leaky integrate-and-fire models (e.g. Izhikevich 2003; Brette and Gerstner 2005; Górski et al 2021) have been developed to approximate electrophysiological realism (see (Brunel and van Rossum 2007), and references therein). A comparison of their performance with more biophysical models has been carried out, among others, in Gerstner and Naud (2009); Izhikevich (2004); Kobayashi (2009). However, despite their effectiveness, nonlinear integrate-and-fire models present additional problems for parameter optimization, as different numerical methods and initial conditions can lead to vastly different results (Kobayashi 2009).

Another class of models, achieving a reasonable compromise between model complexity, biological plausibility, and computational efficiency, is given by *generalized leaky integrate-and-fire* (GLIF) models (e.g. Teeter et al (2018); Wang et al (2014); Jimenez et al (2013)). Differently from the leaky integrate-and-fire (LIF) models, which describe only the membrane potential dynamics (Burkitt 2006), in the GLIF framework the dynamics of the membrane potential and of additional currents (usually representing intrinsic active mechanisms responsible for adaptation or depolarization) is introduced via a system of linear ordinary differential equations coupled with initial and update conditions. These conditions—generally independent from the stimulation current—correspond to the initial conditions assigned to the system after each spike. The GLIF approach is a marked improvement over LIF models and, as shown in a recent work (Teeter et al 2018), provides significant results. However, the parameter optimization procedure was linked to a training set of experimental recordings obtained with a not widely used stimulation protocol, hindering its general applicability to more standard experimental data.

An extended version of this model (therefore called E-GLIF) was introduced in Geminiani et al (2018), to successfully describe cerebellar firing patterns through a constant increment of an adaptation current (independent of the external stimulation) as an update condition. Although it is able to reproduce a variety of cerebellar cell responses with a single set of optimized parameters, this model is unable to capture the main firing properties of CA1 neurons and interneurons. In particular, updating the adaptation current after a spike by adding a constant value often leads to constant inter-spike intervals (ISIs) during a train of action potentials (see e.g. Geminiani et al (2018, 2019)), a rather uncommon condition for CA1 neurons.

To overcome this problem, in this work we propose an adaptive GLIF (A-GLIF) model, where the update conditions depend on the external stimulation current. Starting from a suitable non-dimensionalization procedure to reduce the number of independent model parameters, we carried out a thorough analytical investigation. From a more general point of view, the multiparameter cost function used to optimize this type of model is nonconvex; it is thus important to find ways to constrain the parameter space to maximize the probability to find good solutions in a relatively short time. By studying the system equilibria and their stability as well as the monotonicity properties of the membrane potential, we were able to better constrain the parameter space and obtain quantitative agreement with the number and timing of spikes experimentally observed in 84 CA1 neurons and interneurons in response to a wide range of input currents. These features, used as tuning and validation parameters on a neuron-by-neuron basis, have been proven to be fundamental for effective neuronal models (see e.g. Gerstner and Naud (2009); Jolivet et al (2008)).

In contrast with previous methods (see e.g. Teeter et al (2018)), the A-GLIF model allows to successfully capture the firing features of CA1 neurons and interneurons using only basic, and universally used, stimulation protocols. This approach also allows for an easy implementation of a controlled cloning procedure, increasing the ability of a A-GLIF model to cover the full range of firing patterns observed experimentally and building large networks with more realistic properties (Marasco et al 2023).

The paper is structured as follows: in Sect. 2 we introduce the mathematical model and its nondimensional form, which allows to perform a thorough investigation of the existence and stability of steady-states; this leads to precise constraints on parameters and functional forms for the initial conditions data. The model is then validated under both constant and piecewise constant stimuli. The implications of our results are discussed in Sect. 3, whereas in Sect. 4 we describe the experimental data used as a reference, the statistical analysis, and the optimization procedure.

## Results

### Mathematical Analysis

We start by presenting the mathematical rationale for the A-GLIF model, describing the evolution of the membrane potential *V* and two intrinsic currents, $I_{\textrm{adap}}$ and $I_{\textrm{dep}}$. In particular, the adaptive current $I_{\textrm{adap}}$ implements the activation of the outward currents causing a hyperpolarizing effect, and the depolarizing current $I_{\textrm{dep}}$ representing inward currents. The concurrent dynamic of these currents makes the model able to *a-priori* reproduce several electrophysiological features.

Following (Geminiani et al 2018), we introduce the model defined by the following set of three linear Ordinary Differential Equations (ODEs)

1 $$\begin{aligned} \begin{aligned} \frac{dV}{dt}&= \frac{1}{C_{\textrm{m}}} \left[ \frac{C_\textrm{m}}{\tau _{\textrm{m}}} \left( V-E_{\textrm{L}} \right) -I_{\textrm{adap}}+I_\textrm{dep}+I_{\textrm{stim}} \right] , \\ \frac{dI_{\textrm{adap}}}{dt}&= k_{\textrm{adap}} \left( V-E_{\textrm{L}} \right) - k_2 I_{\textrm{adap}}, \\ \frac{dI_{\textrm{dep}}}{dt}&= -k_1 I_{\textrm{dep}}, \end{aligned} \end{aligned}$$

with all parameters described in Table 1.

All parameters in (1) are positive, except $E_\textrm{L}$, and the injected current $I_{\textrm{stim}}$ (which in general can also be negative). In our simulations, we use either constant or piecewise constant non-negative values.

**Table 1:** List of parameters appearing in Eq. (1), together with their description and units of measurement

Parameter	Description	UM
$E_{\textrm{L}}$	Resting potential	mV
$V_{\textrm{r}}$	Reset potential	mV
$V_{\textrm{th}}$	Threshold potential	mV
$\Delta t_{\textrm{ref}}$	Refractory interval	ms
$I_{\textrm{stim}}$	External stimulation current	pA
$C_{\textrm{m}}$	Membrane capacitance	pF
$\tau _{\textrm{m}}$	Membrane time constant	ms
$I_{\textrm{th}}$	Threshold stimulation current	pA
$k_{\textrm{adap}}$	Adaptation constant	MH$^{-1}$
$k_2$	$I_{\textrm{adap}}$ Decay rate	ms$^{-1}$
$k_1$	$I_{\textrm{dep}}$ Decay rate	ms$^{-1}$

We assume that the neuron is at rest for $t<t_{\textrm{start}}$, i.e., $I_{\textrm{stim}}=0$ and $V=E_{\textrm{L}}$, where $t_{\textrm{start}}$ represents the first time instant at which the stimulation current is different from zero. Moreover, we denote with $I_{\textrm{th}}$ the *threshold current* above which the neuron starts to fire, i.e. we assume that a spike event occurs when, for $I_\textrm{stim}>I_{\textrm{th}}$, the potential *V* reaches the *threshold potential* $V_{\textrm{th}}$.

Starting from the resting condition, the *first spike* for any $I_{\textrm{stim}}>I_{\textrm{th}}$ can be obtained by setting the initial conditions of the Cauchy problem associated to system (1) as follows

2 $$\begin{aligned} \begin{aligned} V (t_{\textrm{start}})&= E_{\textrm{L}}, \\ I_{\textrm{adap}} (t_{\textrm{start}})&= 0, \\ I_{\textrm{dep}} (t_{\textrm{start}})&= I_{\textrm{dep}}^{\textrm{start}}(I_\textrm{stim}-I_{\textrm{th}})\ \theta (I_{\textrm{stim}}-I_{\textrm{th}}), \end{aligned} \end{aligned}$$

where $I_{\textrm{dep}}^{\textrm{start}}$ is a suitable constant and $\theta (I_{\textrm{stim}}-I_{\textrm{th}})$ is the step function defined as

3 $$\begin{aligned} \theta (I_{\textrm{stim}}-I_{\textrm{th}}) = {\left\{ \begin{array}{ll} 1 &{} \text { if } I_{\textrm{stim}}>I_{\textrm{th}}, \\ 0 &{} \text { if } I_{\textrm{stim}}\le I_{\textrm{th}}. \end{array}\right. } \end{aligned}$$

Following the E-GLIF framework, the potential *V* after a spike does not return to the resting value $E_{\textrm{L}}$ but at the *reset potential* $V_{\textrm{r}}$. Then, for any following spike, the initial conditions of each Cauchy problem for (1) are modified according to the following *after-spike update rules*

4 $$\begin{aligned} \begin{aligned} V(t_{\textrm{spk}}^+)&= V_{\textrm{r}}, \\ I_{\textrm{adap}}(t_{\textrm{spk}}^+)&= I_{\textrm{adap}}^0 (t_{\textrm{spk}}^+, I_{\textrm{stim}}),\\ I_{\textrm{dep}}(t_{\textrm{spk}}^+)&= I_{\textrm{dep}}^0, \end{aligned} \end{aligned}$$

where $t_{\textrm{spk}}^+$ is the time instant following the spike time $t_{\textrm{spk}}$, i.e. $t_{\textrm{spk}}^+=t_{\textrm{spk}}+\Delta t_{\textrm{ref}}$, with $\Delta t_{\textrm{ref}} = 2\, \textrm{ms}$ is the *refractory time*, $I_{\textrm{adap}}^0 (t_{\textrm{spk}}^+, I_\textrm{stim})$ is a suitable set of initial values that depend on both the stimulation current $I_{\textrm{stim}}$ and the corresponding spike times, and finally $I_{\textrm{dep}}^0$ is a constant.

We derived an ad-hoc set of update rules for (4) that allowed us to reproduce all the experimentally observed firing behavior of CA1 neurons and interneurons. The rationale for defining the function $I_\textrm{adap}^0 (t_{\textrm{spk}}^+, I_{\textrm{stim}})$ is discussed in Sec. 2.4.

As for any GLIF-type model, all parameters [including those directly related to the initial conditions (3 and 4)] must be determined through an optimization procedure that involves several parameters, especially for the update rules. However, performing a nondimensional analysis of the model allows us to reduce the number of parameters and consequently to obtain a general integral easier to analyze. In particular, following this approach we obtain a stability analysis of the equilibria as a function of two parameters that, together with the monotonicity properties of the membrane potential, can constrain the bidimensional parameter space where to find optimal values, on a neuron-by neuron basis, via an optimization procedure. Moreover, we found a functional form for $I_{\textrm{adap}}^0 (t_{\textrm{spk}}^+, I_{\textrm{stim}})$ that depends only on a few parameters, and can reproduce the entire dynamics experimentally observed in CA1 pyramidal neurons and interneurons.

#### Nondimensional Analysis

Introducing the rescaled variables

5 $$\begin{aligned} {\tilde{t}} = \frac{t}{\tau }, \qquad {\tilde{V}} = - \frac{V}{E_\textrm{L}}, \qquad {\tilde{I}}_{\textrm{adap}} = - \frac{k_2 \, I_\textrm{adap}}{E_{\textrm{L}} \, k_{\textrm{adap}}}, \qquad {\tilde{I}}_{\textrm{dep}} = - \frac{k_2 \, I_{\textrm{dep}}}{E_{\textrm{L}} \, k_{\textrm{adap}}}, \end{aligned}$$

we obtain the following nondimensional version of the system (1) (for simplicity, from now on we will omit the tildes)

6 $$\begin{aligned} \begin{aligned} \frac{dV}{dt}&= \alpha + \beta (I_{\textrm{dep}}-I_{\textrm{adap}})+\delta (1+V), \\ \frac{dI_{\textrm{adap}}}{dt}&= 1-I_{\textrm{adap}}+V, \\ \frac{dI_{\textrm{dep}}}{dt}&= -\gamma I_{\textrm{dep}}, \end{aligned} \end{aligned}$$

where

7 $$\begin{aligned} \alpha =-\frac{I_{\textrm{stim}}}{C_{\textrm{m}} \, E_{\textrm{L}} \, k_2}, \qquad \beta =\frac{k_{\textrm{adap}}}{C_{\textrm{m}} \, k_2^2}, \qquad \gamma =\frac{k_1}{k_2}, \qquad \delta =\frac{1}{k_2 \, \tau _\textrm{m}},\quad \tau =\frac{1}{k_2}. \end{aligned}$$

Assuming $k_1,k_2,k_{\textrm{adap}}>0$, we have consequently $\beta , \, \gamma , \, \delta > 0$, while the sign of $\alpha$ depends on whether $I_{\textrm{stim}}$ is positive or negative.

At this point, in order to simplify the analysis we impose $\beta = \gamma$, i.e.

8 $$\begin{aligned} k_{\textrm{adap}}=C_{\textrm{m}} \,k_1 \, k_2. \end{aligned}$$

This assumption implies that $k_1, k_2$ and $k_{\textrm{adap}}$ are not independent from each other. In particular, condition (8) allows to link the ($V-$dependent) $I_{\textrm{adap}}$ dynamics to both a specific cell property ($C_{\textrm{m}}$) and the dynamics of $I_\textrm{dep}$. Introducing without loss of generality a scaling constant $K>0$ such that

9 $$\begin{aligned} k_2 = -\frac{K}{C_{\textrm{m}} \, E_{\textrm{L}}}, \end{aligned}$$

the nondimensional system (6) becomes

10 $$\begin{aligned} \begin{aligned} \frac{dV}{dt}&= \alpha + \beta (I_{\textrm{dep}}-I_{\textrm{adap}})+\delta (1+V), \\ \frac{dI_{\textrm{adap}}}{dt}&= 1-I_{\textrm{adap}}+V, \\ \frac{dI_{\textrm{dep}}}{dt}&= -\beta I_{\textrm{dep}}, \end{aligned} \end{aligned}$$

where

11 $$\begin{aligned} \alpha = \frac{I_{\textrm{stim}}}{K}, \qquad \beta = \frac{k_1}{k_2}, \qquad \delta =\frac{1}{k_2\, \tau _{\textrm{m}}}. \end{aligned}$$

We remark that all model parameters $\alpha ,\beta ,\delta$, and $\tau$ depend on the dimensional constant quantities *K*, $k_1$, $k_2$, $\tau _{\textrm{m}}$.

The nondimensional parameters in Eq. (10) can be interpreted as follows: $\alpha$ represents the scaled injected current; $\beta$ is the ratio between the decay rate of the depolarization current ($k_1$) and the adaptation current ($k_2$); $\delta$ represents the effective rate of change for the membrane potential, caused by the decay rate of the adaptation current ($k_2$) and the intrinsic system time constant ($\tau _{\textrm{m}}$).

Similarly, the dimensionless initial conditions (2) and (4) assume the following forms, respectively,

12a $$\begin{aligned}&V(t_{\textrm{start}})=-1,{} & {} V(t_{\textrm{spk}}^+)=-\frac{V_{\textrm{r}}}{E_{\textrm{L}}},\end{aligned}$$

12b $$\begin{aligned}&I_{\textrm{adap}}(t_{\textrm{start}})=0,{} & {} I_{\textrm{adap}}(t_\textrm{spk}^+)=I_{\textrm{adap}}^0(t_{\textrm{spk}}^+,I_{\textrm{stim}}),\end{aligned}$$

12c $$\begin{aligned}&I_{\textrm{dep}}(t_{\textrm{start}})=I_{\textrm{dep}}^{\textrm{start}}(I_\textrm{stim}-I_{\textrm{th}})\theta (I_{\textrm{stim}}-I_{\textrm{th}}),{} & {} I_\textrm{dep}(t_{\textrm{spk}}^+)=I_{\textrm{dep}}^0, \end{aligned}$$

where all time variables have been rescaled by means of $\tau =1/k_2$ [cf. Eq. (5)].

#### General Integral

Assuming a constant stimulation current $I_{\textrm{stim}}$, the linear nature of the system (10), depending only on $\alpha ,\beta ,\delta$, permits to obtain the explicit form of the solutions in terms of the following initial data

13 $$\begin{aligned} V(t_0):= V^0, \quad I_{\textrm{adap}}(t_0):= I_{\textrm{adap}}^0, \quad I_{\textrm{dep}}(t_0):=I_{\textrm{dep}}^0. \end{aligned}$$

In detail, we obtain

14 $$\begin{aligned} V(t)= & {} -I_{\textrm{adap}}^0 \beta {\mathcal {H}}_1+I_{\textrm{dep}}^0\frac{\beta \left[ (\beta -1) \left( {\mathcal {H}}_2-2 e^{\beta (t_0-t)}\right) \right] +{\mathcal {H}}_1 \left[ (\beta -1) (\delta +1)+2 \beta \right] }{2 \left( \beta ^2+(\beta -1) \delta \right) }\nonumber \\{} & {} +\frac{V_0}{2} \left[ (\delta +1) {\mathcal {H}}_1+{\mathcal {H}}_2\right] +\frac{{\mathcal {H}}_1}{2} \left[ \frac{\alpha (\beta -1)}{\beta -\delta }+\alpha +\delta +1\right] -\frac{({\mathcal {H}}_2-2) (\alpha -\beta +\delta )}{2 (\beta -\delta )},\nonumber \\ I_{\textrm{adap}}(t)= & {} \frac{1}{2} I_{\textrm{adap}}^0 \left[ {\mathcal {H}}_2-(\delta +1) {\mathcal {H}}_1\right] +I_\textrm{dep}^0\frac{\beta }{2}\left[ \frac{2 e^{\beta (t_0-t)}+{\mathcal {H}}_1 (2 \beta +\delta -1)-{\mathcal {H}}_2}{ \beta ^2+(\beta -1) \delta }\right] \nonumber \\{} & {} +V_0 {\mathcal {H}}_1-\frac{\alpha }{2}\left[ \frac{(1-\delta ) {\mathcal {H}}_1+{\mathcal {H}}_2-2}{\beta -\delta }\right] +{\mathcal {H}}_1, \nonumber \\ I_{\textrm{dep}}(t)= & {} I_{\textrm{dep}}^0 e^{-\beta (t-t_0)}, \end{aligned}$$

where

15 $$\begin{aligned} {\mathcal {A}}=(\delta +1)^2-4 \beta ,{} & {} \quad {\mathcal {B}}= -\frac{1}{2} \left( \sqrt{{\mathcal {A}}}-\delta +1\right) (t-t_0),\quad {\mathcal {C}}=\sqrt{{\mathcal {A}}} (t-t_0), \end{aligned}$$

16 $$\begin{aligned}{} & {} {\mathcal {H}}_1= \frac{e^{{\mathcal {B}}} \left( e^{{\mathcal {C}}}-1\right) }{\sqrt{{\mathcal {A}}}},\quad {\mathcal {H}}_2= e^{{\mathcal {B}}} \left( e^{{\mathcal {C}}}+1\right) . \end{aligned}$$

This result allows to simply evaluate, in subthreshold dynamics, the value of *V*(*t*) at any time avoiding the use of numerical integration methods to advance the ODE system.

### Equilibria and Stability Analysis

A crucial step, in obtaining a better and faster model optimization, is to limit as much as possible the parameters’ space reproducing the reference traces. For this purpose, we performed a stability analysis of the equilibria. System (10) admits two types of steady-state solutions $( V^*,\, I_{\textrm{adap}}^*,\, I_\textrm{dep}^*)$, which are classified below.

- For $\alpha =0$ and $\beta =\delta \ne 0$ there are infinite equilibria of the following form
  17 $$\begin{aligned} E_0:= \left( {\bar{V}},\, 1+{\bar{V}},\, 0 \right) , \end{aligned}$$
  where ${\bar{V}}$ represents the steady-state value of the (non dimensional) membrane potential *V*. Considering ${\bar{V}}=-1$, we have that Eq. (17) reduces to $E_0^* = \left( -1,\, 0,\, 0 \right)$.
- For $\alpha \ne 0$ or $\beta \ne \delta$ there is a unique equilibrium given by
  18 $$\begin{aligned} E_1:= \left( \frac{\alpha }{\beta - \delta }-1,\, \frac{\alpha }{\beta - \delta },\, 0 \right) . \end{aligned}$$

Because the system (10) is linear, the stability properties of the steady-states are global and can be determined by studying the eigenvalue problem of the associated Jacobian matrix

19 $$\begin{aligned} M:= \begin{pmatrix} \delta &{} -\beta &{} \beta \\ 1 &{} -1 &{} 0 \\ 0 &{} 0 &{} -\beta \\ \end{pmatrix}. \end{aligned}$$

The matrix *M* admits the following three eigenvalues (independent from $\alpha$ and therefore from $I_{\textrm{stim}}$)

20 $$\begin{aligned} \begin{aligned} \lambda _1&= -\beta , \\ \lambda _2&= \frac{1}{2} \left( \delta - 1 + \sqrt{\Delta } \right) , \\ \lambda _3&= \frac{1}{2} \left( \delta - 1 - \sqrt{\Delta } \right) , \end{aligned} \end{aligned}$$

with $$\Delta = (1+\delta )^2-4 \beta $$.

**Fig. 1:**
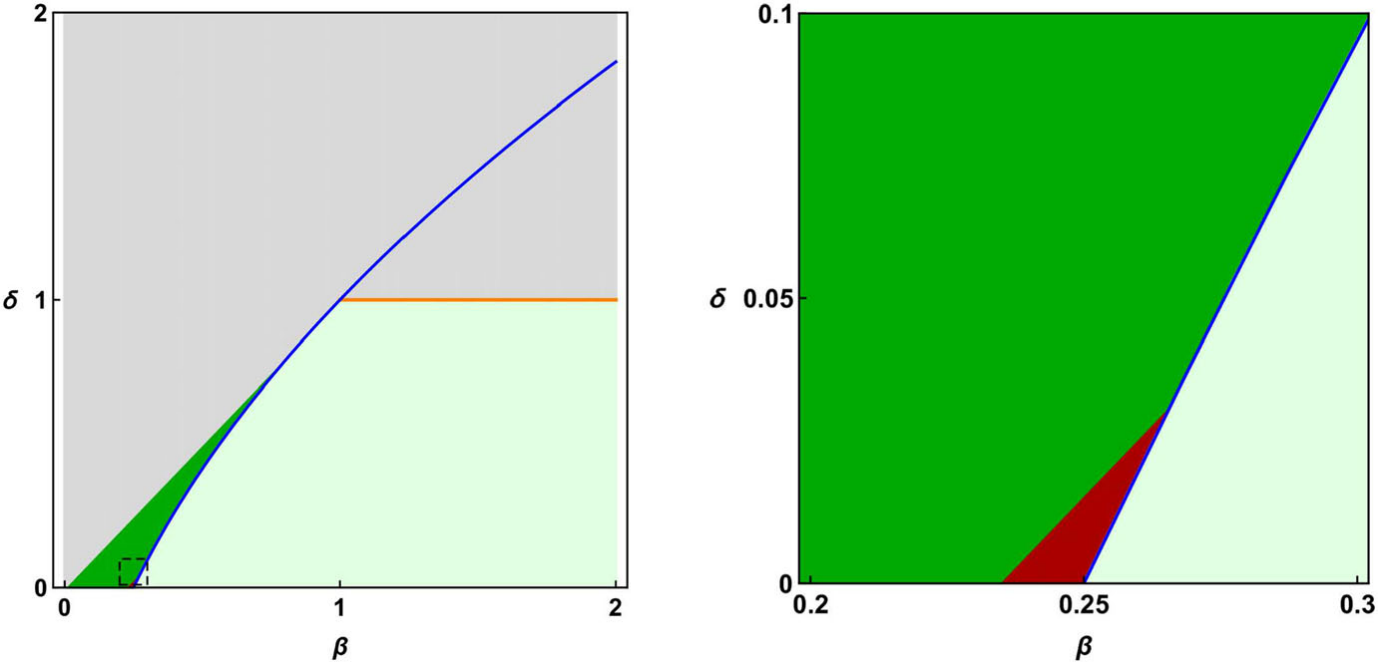
Stability analysis. Left: stability regions for the steady-state $E_1$ in the $(\beta ,\,\delta )$ parameter space. The green areas represent $(\beta ,\,\delta )$ values for which $E_1$ is asymptotically stable (dark green: real eigenvalues, light green: complex eigenvalues—see (21) and (23), respectively). The blue curve defines the points where $\Delta =0$, whereas the orange line represents simple stability region for $E_1$ [see Eq. (24)]. The gray area covers the instability range given in Eqs. (22), (25). The dashed square indicates the zoomed area in the right panel where the dark red area defines the subregion of the asymptotic stability regime provided by Eq. (28) for $\alpha _{\textrm{th}}=0.0665$ and ${\tilde{V}}_{\textrm{th}}=-0.717$, corresponding to the computational NEURON model

When considering the steady-states in Eq. (17), the eigenvalues in (20) reduce to $\lambda _1=-\delta$, $\lambda _2=0$, and $\lambda _3=\delta -1$, and the Jacobian matrix is diagonalizable. We hence have that these equilibria are stable for $\delta \le 1$ and unstable for $\delta > 1$. As for the steady-state in Eq. (18), we observe that $\beta > 0$ implies $\lambda _1 < 0$; therefore, the stability properties depend only on the sign of $\lambda _2$ and $\lambda _3$. In particular, we have the following cases (summarized in Fig. 1):

- When $\Delta \ge 0$, the two eigenvalues $\lambda _2,\lambda _3$ are real.
  - Both eigenvalues are negative - hence the steady state in (18) is asymptotically stable - if and only if
    21 $$\begin{aligned} \delta< \beta \le \frac{1}{4} (1+\delta )^2 \quad \text { with } 0< \delta < 1. \end{aligned}$$
  - On the other hand, at least one between $\lambda _2$ and $\lambda _3$ is positive (therefore implying instability of the steady state) if and only if
    22 $$\begin{aligned} \beta< \delta ,\quad 0 < \delta \le 1,\quad \quad \text { or }\quad \quad \beta \le \frac{1}{4}(1+\delta )^2, \quad \delta > 1. \end{aligned}$$
- When $\Delta < 0$, $\lambda _2$ and $\lambda _3$ are complex conjugates, and therefore there is an oscillatory dynamics around the steady-state (18).
  - The oscillations are damped (hence implying asymptotic stability of the steady-state) when the real part of $\lambda _2$ and $\lambda _3$ is negative, namely if and only if
    23 $$\begin{aligned} \beta > \frac{1}{4} (1+\delta )^2 \quad \text { with } 0< \delta < 1. \end{aligned}$$
  - The oscillations are sustained (i.e. we have simple stability) when the real part of $\lambda _2, \, \lambda _ 3$ is zero and their algebraic as well as geometric multiplicity is equal to one. This occurs if and only if
    24 $$\begin{aligned} \beta >1 \;\text { and }\; \delta = 1. \end{aligned}$$
  - Finally, at least one between $\lambda _2$ and $\lambda _3$ has a positive real part [leading to instability of the steady-state (18)] if and only if
    25 $$\begin{aligned} \beta> \frac{1}{4}(1+\delta )^2 \quad \text { with }\; \delta > 1. \end{aligned}$$

The instability range in (25) can be linked to a *rebound spiking effect*, i.e. a phenomenon observed in a neuron during the repolarization phase following a hyperpolarized condition (Ascoli et al 2010). Its occurrence is mediated by the dynamic interplay among several ionic currents, and it is rarely observed in CA1 principal neurons under physiological conditions. For this reason, this effect will not be taken into account in this work, and for all our cases we will impose condition (21).

These results, and in particular condition (21) (which will ensure that a cell will never reach $V_{\textrm{th}}$ for $I_{\textrm{stim}}<I_{\textrm{th}}$), imply that the numerical values of $\beta ,\delta$ reproducing the pyramidal cells and interneurons’ firing patterns can be limited to the dark green region in Fig. 1, where we show the type of stability as a function of $\delta$ and $\beta$. Their set of values may be further restricted in a much smaller region, according to the specific firing properties of a given cell. This is shown by the dark red region in the right panel of Fig. 1, illustrating the region of asymptotic stability covering the firing properties of the NEURON model (see Sect. 4).

### Parameter constraints and initial data sequences

Despite the linearity of the equations, the A-GLIF model can reproduce a wide range of physiological firing patterns like bursting, non adapting, and continuous adapting (Migliore et al 2018), provided that the initial data sequences, $I_{\textrm{dep}}^0$ and $I_{\textrm{adap}}^0$, are suitably chosen. For this purpose, a fundamental step is to carry out an analytical study of the spiking properties as a function of the model parameters.

#### Constraints on the parameters $\beta$ and $\delta$

Here we derive the parameters’ constraints guaranteeing that the cell will not spike for $0<I_{\textrm{stim}}< I_{\textrm{th}}$. To this aim, we exploit the asymptotic stability results presented in Sect. 2.2 to impose that when the external stimulation current is lower than the threshold current $I_{\textrm{th}}$ the *V*-component of the steady-state $E_1$ lies below the spiking threshold. Owing to the asymptotic stability conditions (21), imposing that

26 $$\begin{aligned} \frac{\alpha }{\beta -\delta }-1<{\tilde{V}}_{\textrm{th}},\quad \forall I_\textrm{stim}: I_{\textrm{stim}}< I_{\textrm{th}}, \end{aligned}$$

where ${\tilde{V}}_{\textrm{th}}=-V_{\textrm{th}}/E_{\textrm{L}}$ is the nondimensional form of the threshold potential $V_{\textrm{th}}$, we obtain the following constraints^1^

27 $$\begin{aligned} \alpha< \frac{(1+{\tilde{V}}_{\textrm{th}})(\delta -1)^2}{4}, \quad \frac{\alpha }{1+{\tilde{V}}_{\textrm{th}}}+\delta< \beta \le \frac{1}{4} (\delta +1)^2,\quad 0< \delta < 1. \end{aligned}$$

In particular, for $I_{\textrm{stim}}=I_{\textrm{th}}$ we obtain

28 $$\begin{aligned} \alpha _{\textrm{th}}< \frac{(1+{\tilde{V}}_{\textrm{th}})(\delta -1)^2}{4}, \quad \frac{\alpha _{\textrm{th}}}{1+{\tilde{V}}_{\textrm{th}}}+\delta< \beta \le \frac{1}{4} (\delta +1)^2,\quad 0< \delta < 1, \end{aligned}$$

where $\alpha _{\textrm{th}}=I_{\textrm{th}}/K$, and the corresponding dimensional constraints can be written as

29a $$\begin{aligned}&K > -\frac{C_{\textrm{m}} \, E_{\textrm{L}}}{\tau _{\textrm{m}}} + \frac{2 E_\textrm{L}}{E_{\textrm{L}}-V_{\textrm{th}}} \left[ I_{\textrm{th}}+\sqrt{I_\textrm{th}^2-\frac{I_{\textrm{th}}C_{\textrm{m}} (V_{\textrm{th}}-E_{\textrm{L}})}{\tau _\textrm{m}} }\right] , \end{aligned}$$

29b $$\begin{aligned}&\frac{K}{E_{\textrm{L}} \tau _{\textrm{m}}} \left[ \frac{I_{\textrm{th}}\tau _\textrm{m}}{C_{\textrm{m}}(E_{\textrm{L}}-V_{\textrm{th}})} - 1 \right] < k_{\textrm{adap}} \le \frac{(C_{\textrm{m}} \, E_{\textrm{L}}-K \, \tau _{\textrm{m}})^2}{4 C_{\textrm{m}} \, E_{\textrm{L}}^2 \, \tau _{\textrm{m}}^2}. \end{aligned}$$

#### Constraints on the initial data $I_{\textrm{dep}}^0$ and $I_{\textrm{adap}}^0$

A natural condition to impose (when not restrictive, see Sect. 2.5.1) is that *V* is an increasing function of *t* for any positive stimulation current $I_{\textrm{stim}}> I_{\textrm{th}}$. Hence, $I_{\textrm{dep}}^0$ and $I_{\textrm{adap}}^0$ after a spike must satisfy the following condition [see Eq. (12)]

30 $$\begin{aligned} I_{\textrm{adap}}^0 < \frac{\alpha }{\beta }+I_\textrm{dep}^0+\frac{\delta }{\beta } (1+V^0), \end{aligned}$$

where $V^0=-1$ or $V^0=-V_r/E_{\textrm{L}}$ for the first or after the first spike event, respectively.

Furthermore, in view of Eqs. (14) and (21), it is easy to prove that *V* is a decreasing function with respect to $I_{\textrm{adap}}^0$ when the other initial data are fixed. In fact, we have

31 $$\begin{aligned} \frac{d V}{d I_{\textrm{adap}}^0}=\frac{\beta e^{\frac{1}{2} \delta (t-t_0)}}{\sqrt{(\delta +1)^2-4 \beta }}\left[ 1- e^{\sqrt{(\delta +1)^2-4 \beta } (t-t_0)}\right] <0. \end{aligned}$$

In contrast, under the constraints (21), it can be numerically proved that

32 $$\begin{aligned} \frac{d V}{d I_{\textrm{dep}}^0}>0, \end{aligned}$$

for any fixed $I_{\textrm{adap}}^0$ and $V^0$. Typical values for the derivative in Eq. (32) are shown in Suppl. Fig. 1 for a range of $\delta$ values.

Considering that the model is autonomous for any constant stimulation current $I_{\textrm{stim}}$, a nonuniform sequence of ISIs can be obtained if and only if at least one of the initial conditions for the update rules must change after each spike. In particular, owing to Eq. (31), if we fix the initial values of $V^0$ and $I_{\textrm{dep}}^0$, increasing values of $I_{\textrm{adap}}^0$ will result in increasing ISIs, and vice versa.

### Functional form of $I_{\textrm{adap}}^0 (t_{\textrm{spk}}^+, I_{\textrm{stim}})$ sequence

**Fig. 2:**
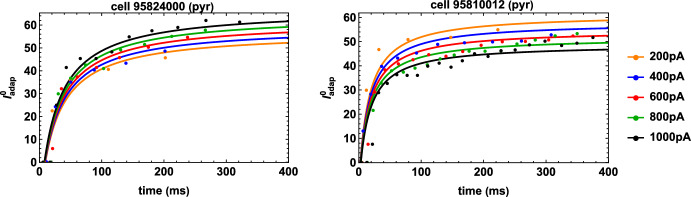
Monod-type functions (33) (continuous lines) interpolating the initial data sequences $I_{\textrm{adap}}^0(t_\textrm{spk}, I_{\textrm{stim}})$ (dots) for the pyramidal cells 95824000 (left panel) and 95810012 (right panel). The colors indicate the intensity of the constant stimulation currents (color figure online)

In order to reproduce the experimentally observed spike-times as a function of the stimulation current $I_{\textrm{stim}}$, an optimization procedure must thus find a sequence of $I_{\textrm{adap}}^0 (t_\textrm{spk}^+, I_{\textrm{stim}})$, where $I_{\textrm{stim}} \in \left[ I_\textrm{stim}^{\textrm{min}}, I_{\textrm{stim}}^{\textrm{max}} \right]$. The process can be very efficient and accurate, if it is implemented following the constraints discussed in the previous sections. For our set of reference data, $I_{\textrm{stim}}^{\textrm{min}}$ and $I_{\textrm{stim}}^\textrm{max}$ are (neuron dependent) quantities which define the minimum and maximum stimulation current eliciting spikes (200–1000pA for most of our reference traces). The set of $I_{\textrm{adap}}^0$ values found by the optimization procedure (see Sect. 4) for two typical pyramidal neurons is shown in Fig. 2, and represented with colored circles. Note that the set of values for each current may increase (e.g. cell 95824000) or decrease (as for cell 95810012) as a function of the stimulation strength. Although our model can be applied to any type of neurons, we are interested in reproducing the main electrophysiological features of CA1 pyramidal neurons and interneurons. The optimization results suggested that the $I_{\textrm{adap}}^0 (t_{\textrm{spk}}^+, I_{\textrm{stim}})$ sequence for any given cell can be interpolated by a Monod-type function as (Monod (1949))

33 $$\begin{aligned} I_{\textrm{adap}}^0(t_{\textrm{spk}}^+, I_{\textrm{stim}}):= c+\frac{a \, e^{b \, I_{\textrm{stim}}} \, (t_{\textrm{spk}}^+-t_{\textrm{start}})}{d + (t_\textrm{spk}^+-t_{\textrm{start}})}, \quad \forall I_{\textrm{stim}} \in \left[ I_\textrm{stim}^{\textrm{min}}, I_{\textrm{stim}}^{\textrm{max}} \right] , \end{aligned}$$

where *a*, *b*, *c*, *d* are constants, and $t_{\textrm{start}}$ is the last instant in which $I_{\textrm{stim}}=0$ or $I_{\textrm{stim}}\le I_{\textrm{th}}$.

The function (33) is defined for all $d+(t_\textrm{spk}^{\textrm{first}}-t_{\textrm{start}}) \ne 0$, where $t_{\textrm{spk}}^\textrm{first}$ is the time of the first spike event for the current $I_\textrm{stim}$. For sake of simplicity, we assume that $d \ge 0$.

Let us define $\chi := t_{\textrm{spk}}^+-t_{\textrm{start}}$. This positive quantity allows us to rewrite Eq.  (33) as

34 $$\begin{aligned} I_{\textrm{adap}}^0(\chi , I_{\textrm{stim}}):= c+\frac{a \, e^{b \, I_\textrm{stim}} \, \chi }{d + \chi }. \end{aligned}$$

In the case of constant $I_{\textrm{stim}}$, we have that $t_\textrm{start}=0$ is the last instant where $I_{\textrm{stim}}=0$; consequently, for any fixed value of $I_{\textrm{stim}}>0$ we obtain $\chi = t_\textrm{spk}^+ \in \left[ t_{\textrm{spk}}^{\textrm{first}}\left( I_{\textrm{stim}}\right) ,t_{\textrm{spk}}^{\mathrm{last-1}}\left( I_{\textrm{stim}}\right) \right]$. On the other hand, when the stimulation current is varying, the value $t_{\textrm{start}}$ is updated to the last instant in which $I_\textrm{stim}=0$ or $I_{\textrm{stim}}\le I_{\textrm{th}}$; therefore, we have more generally $\chi \in \left[ t_{\textrm{spk}}^{\textrm{first}}\left( I_\textrm{stim}\right) -t_{\textrm{start}},t_{\textrm{spk}}^{\mathrm{last-1}}\left( I_{\textrm{stim}}\right) -t_{\textrm{start}}\right]$. The constraints on the constants *a*, *b*, *c*, *d* in Eq.  (34) are derived by imposing the two following requirements on the Monod function:

1. The Monod function must be positive, i.e.
  35 $$\begin{aligned} I_{\textrm{adap}}^{0}(\chi ,I_{\textrm{stim}})\ge 0,\quad \forall I_\textrm{stim}>I_{\textrm{th}}>0,\; \forall \chi \in \left[ t_{\textrm{spk}}^\textrm{first}\left( I_{\textrm{stim}}\right) ,t_{\textrm{spk}}^{\mathrm{last-1}}\left( I_{\textrm{stim}}\right) \right] .\nonumber \\ \end{aligned}$$
  We observe that the interval $\left[ t_{\textrm{spk}}^{\textrm{first}}\left( I_{\textrm{stim}}\right) ,t_{\textrm{spk}}^{\mathrm{last-1}}\left( I_\textrm{stim}\right) \right]$ is a priori not known for all $I_\textrm{stim}>I_{\textrm{th}}$, as the only information available regards the experimental data within the interval $\left[ I_{\textrm{stim}}^\textrm{min},I_{\textrm{stim}}^{\textrm{max}}\right]$. It is then mathematically more convenient to determine the parameter conditions such that Eq.  (35) holds $\forall \chi > 0$.
2. Assuming that, $\forall \chi \in \left[ t_{\textrm{spk}}^{\textrm{first}}\left( I_{\textrm{stim}}\right) ,t_{\textrm{spk}}^\mathrm{last-1}\left( I_{\textrm{stim}}\right) \right]$ and $\forall I_\textrm{stim}\in \left[ I_{\textrm{stim}}^{\textrm{min}},I_{\textrm{stim}}^\textrm{max}\right]$, we have
  36 $$\begin{aligned} I_{\textrm{adap}}^{0}(\chi ,I_{\textrm{stim}})<\frac{\alpha }{\beta }+I_{dep}^{0}+\frac{\delta }{\beta }\left( 1+V^{0}\right) =: H(I_{\textrm{stim}}), \end{aligned}$$
  in order to ensure Eq. (30), the following inequality must hold
  37 $$\begin{aligned} \begin{aligned} I_{\textrm{adap}}^{0}(\chi ,I_{\textrm{stim}})<H(I_{\textrm{stim}})\quad&\forall \chi \in \left[ t_{\textrm{spk}}^{\textrm{first}}\left( I_{\textrm{stim}}\right) ,t_{\textrm{spk}}^{\mathrm{last-1}}\left( I_{\textrm{stim}}\right) \right] ,\\ \quad&\forall I_{\textrm{stim}}>I_{th}>0. \end{aligned} \end{aligned}$$

#### Remark 1

Equation (34) defines a sequence of constant ISIs with $I_{\textrm{adap}}^0$ independent from $I_{\textrm{stim}}$ if $a=0$ and $c \ge 0$. In this case, in fact, we obtain $I_{\textrm{adap}}^0 \equiv c$. Analogously, Eq. (34) defines a sequence of constant ISIs with $I_{\textrm{adap}}^0$ dependent from $I_{\textrm{stim}}$ if $d=0$ and $c+a\exp (b \, I_{\textrm{stim}})>0$. In this case, in fact, we obtain $I_{\textrm{adap}}^0(I_{\textrm{stim}}) = c+a\exp (b \, I_{\textrm{stim}})$.

Under this condition, the model will thus support suprathreshold oscillations (constant ISIs) whereas, analogously to any other model based on LIF equations, it cannot support subthreshold oscillations when $I_{\textrm{stim}}$ is constant. As the firing dynamics we aim to describe involve nonconstant ISIs, from now on we will exclude the two scenarios described in Remark 1 by assuming that $a, d \ne 0$. Under these assumptions, we obtain $d>0$.

We are hence able to derive several monotonicity properties for the Monod function $I_{\textrm{adap}}^{0}(\chi ,I_{\textrm{stim}})$ in (34), which we summarize in the following result.

#### Proposition 1

Let us assume $a \ne 0$ and $d > 0$. The following properties hold:

1. The Monod function $I_{\textrm{adap}}^{0}(\chi ,I_{\textrm{stim}})$ is monotonic with respect to $\chi$; in particular, it is monotonically increasing (resp. decreasing) if $a>0$ (resp. $\hbox {a}<0$).
2. The Monod function $I_{\textrm{adap}}^{0}(\chi ,I_{\textrm{stim}})$ is monotonic with respect to $I_{\textrm{stim}}$; in particular, it is monotonically increasing (resp. decreasing) if $a b>0$ (resp. $a b<0$).
3. The limit values for the Monod function $I_{\textrm{adap}}^{0}(\chi ,I_{\textrm{stim}})$ with respect to $\chi$ are given by
  38 $$\begin{aligned} \begin{aligned} \lim \limits _{\chi \rightarrow 0^{+}}I_{\textrm{adap}}^{0}(\chi ,I_{\textrm{stim}})&=c, \\ \lim \limits _{\chi \rightarrow +\infty }I_{\textrm{adap}}^{0}(\chi ,I_\textrm{stim})&=c+a\exp (b \, I_{\textrm{stim}}) =: P(I_{\textrm{stim}}), \end{aligned} \end{aligned}$$
  where $P(I_{\textrm{stim}})$ defines the asymptotic plateau of $I_\textrm{adap}^{0}(\chi ,I_{\textrm{stim}})$.
4. The plateau $P(I_{\textrm{stim}})$ of the Monod function $I_\textrm{adap}^{0}(\chi ,I_{\textrm{stim}})$ is monotonic with respect to $I_\textrm{stim}$; in particular, it is monotonically increasing (resp. decreasing) if $a b >0$ (resp. $a b < 0$).
5. The threshold function $H(I_{\textrm{stim}})$ in Eq. (37) is monotonically increasing.

#### Proof

We split the proof in different steps referring to the corresponding results above.

1. We have that
  $$\begin{aligned} \frac{d}{d\chi }I_{\textrm{adap}}^{0}(\chi ,I_{\textrm{stim}})=\frac{ad\exp (b \, I_{\textrm{stim}})}{\left( d+\chi \right) ^{2}}. \end{aligned}$$
  This quantity is positive (resp. negative) if $a>0$ (resp.  $a<0$).
2. The derivative of $I_{\textrm{adap}}^{0}(\chi ,I_{\textrm{stim}})$ with respect to $I_{\textrm{stim}}$ is given by
  $$\begin{aligned} \frac{d}{dI_{\textrm{stim}}}F(\chi ,I_{\textrm{stim}})=\frac{ab\exp (b \, I_{\textrm{stim}})\chi }{d+\chi }. \end{aligned}$$
  This quantity is positive (resp. negative) if $a b>0$ (resp. $a b<0$).
3. The result derives from direct calculation.
4. The derivative of $P(I_{\textrm{stim}})$ with respect to $I_{\textrm{stim}}$ is given by
  $$\begin{aligned} \frac{d}{dI_{\textrm{stim}}}P(I_{\textrm{stim}})=ab\exp (b \, I_{\textrm{stim}}). \end{aligned}$$
  This quantity is positive (resp. negative) if $a b>0$ (resp. $a b<0$).
5. Recalling Eq. (11), we have that the derivative of $H(I_{\textrm{stim}})$ is given by
  $$\begin{aligned} \frac{d}{dI_{\textrm{stim}}}H(I_{\textrm{stim}})=\frac{1}{K\beta }, \end{aligned}$$
  which is positive for all values of $I_{\textrm{stim}}$.

$$\square $$

Proposition 1 allows us to derive the sufficient conditions on the parameters *a*, *b*, *c*, *d* which ensure that Eq. (35) is satisfied for any $\chi > 0$.

#### Proposition 2

The Monod function $I_{\textrm{adap}}^{0}(\chi ,I_{\textrm{stim}})$ is positive [i.e. satisfies Eq. (35)] if the following conditions hold for all $I_{\textrm{stim}}>0$ and $\chi > 0$

(i): either $$a>0,\quad c\ge 0,\quad \forall b$$,
(ii): or $$a<0,\quad b<0,\quad c\ge -a$$.

#### Proof

From Proposition 1 we have that, for all $\chi > 0$, the Monod function $I_{\textrm{adap}}^{0}(\chi ,I_{\textrm{stim}})$ is bounded as follows:39Moreover, we observe that the plateau $P(I_{\textrm{stim}})$ is positive for any $I_{\textrm{stim}}>0$ if

- for $$a>0$$, if
  - either $$c \ge 0, \quad \forall b$$,
  - or $$b>0, \quad -a \le c < 0$$;
- for $a<0$, if $b<0$ and $c \ge -a$.

The combination of these observations proves our claim. $\square$

The conditions defined in Proposition 2 ensure the positivity of the Monod function for all $\chi > 0$ and $I_{\textrm{stim}}>0$. However, in the application of our optimization procedure we only require that the Monod function remains positive as long as the neuron fires; hence, the interpolation of the experimental data for $I_{\textrm{adap}}^{0}(t_{\textrm{spk}}^+,I_{\textrm{stim}})$ may lead to Monod functions for which $c<0$ when $a>0$ or $P(I_{\textrm{stim}})<0$ for $a < 0$. The above considerations still allow us to derive necessary conditions which ensure the positivity of the Monod function in the experimental range – i.e. for any $I_{\textrm{stim}}\in \left[ I_\textrm{stim}^{\textrm{min}},I_{\textrm{stim}}^{\textrm{max}}\right]$ – on the interval $\left[ t_{\textrm{spk}}^{\textrm{first}}\left( I_{\textrm{stim}}\right) ,t_\textrm{spk}^{\mathrm{last-1}}\left( I_{\textrm{stim}}\right) \right]$, given by

(i) $$a>0\Rightarrow P(I_{\textrm{stim}})>0$$,
(ii) $$a<0\Rightarrow c>0$$.

By simultaneously fitting, for each neuron, the set of $I_\textrm{adap}^0$ values for all experimental currents using Eq. (33) (solid curves in Fig. 2), we obtain a function with which we can now predict the spike times of a given neuron for any constant current injection in the interval $\left[ I_{\textrm{stim}}^{\mathrm{{min}}}, \, I_{\textrm{stim}}^{\mathrm{{max}}}\right]$.

**Fig. 3:**
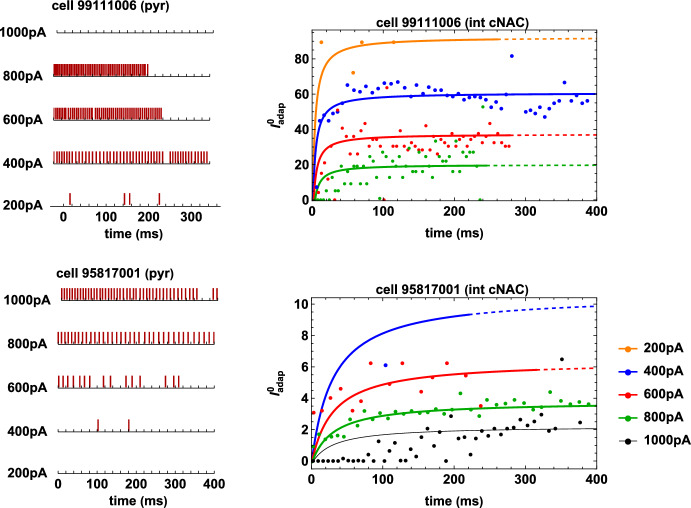
Examples of Monod function with firing block fitting the sequence of optimized $I_{\textrm{adap}}^0$. Left: experimental raster plots. The upper panels correspond to the interneuron cNAC 99111006 [see Eq. (45)], whereas the lower panels are valid for the interneuron cNAC 95817001 [see Eq. (47)]. Right: Fitted Monod functions for the interneurons cNAC 99111006 (upper panel) and cNAC 95817001 (lower panel); the line is continuous until the firing block is activated, then it becomes dashed (color figure online)

This implementation results in a model neuron that will keep firing as long as the current injection is above $I_{\textrm{th}}$ in the interval $\left[ I_{\textrm{stim}}^{\mathrm{{min}}}, \, I_{\textrm{stim}}^\mathrm{{max}}\right]$. However, in several cases, experimental traces show a sudden block of firing, long before the end of the stimulation, as shown in the left panels of Fig. 3 for two typical interneurons. From a biophysical viewpoint, a *firing block* is considered to occur when a neuron stops firing and never recovers from this state.^2^ To reproduce this condition for any neuron and any stimulation current $I_{\textrm{stim}}$, we implemented a *Monod block procedure* as follows. First, let us assume that for the injected current $I_{\textrm{stim}}$ a firing block occurs when the following condition holds

40 $$\begin{aligned} t_{\textrm{spk}}^{\mathrm{\mathrm last}}(I_{\textrm{stim}})+2\,ISI_\mathrm{\mathrm last}(I_{\textrm{stim}})<T, \end{aligned}$$

where $t_{\textrm{spk}}^{\mathrm{\mathrm last}}$ and $ISI_{\mathrm{\mathrm last}}$ are the time and the ISI of the last spike event for the current $I_\textrm{stim}$, respectively, and $[t_{\textrm{start}},T]$ is the stimulation interval. In other words, when Eq. (40) is verified, we assume that the neuron has entered a firing block state. This choice is consistent with the experimentally observed average value of approximately 2 for the maximum ratio between late and early action potential inter-spike intervals in a train [(see (Scorza et al 2011)]. In the left panels of Fig. 3 we see that for the interneuron cNAC 99111006 (upper panel) firing blocks occur at 237.02ms for $I_{\textrm{stim}}=200$pA, at 276.83ms for $I_\textrm{stim}=600$pA, and at 240.42ms for $I_{\textrm{stim}}=800$pA, whereas the neuron keeps firing for $I_{\textrm{stim}}=400$pA and never fires for $I_{\textrm{stim}}=1000$pA. Coherently with our definition, Eq. (40) is satisfied in this case for $I_\textrm{stim}=200,600,800$pA. Analogously, for the interneuron cNAC 95817001 (lower panel) we observe firing blocks at 199.72ms for $I_\textrm{stim}=400$pA and at 303.43ms for $I_{\textrm{stim}}=600$pA, whereas the neuron fires for the entire length of the recording for $I_\textrm{stim}=800, 1000$pA and never fires for $I_{\textrm{stim}}=200$pA. In this case, Eq. (40) is satisfied for $I_\textrm{stim}=400,600$pA. We thus expect firing blocks to occur in the two intervals of stimulation currents $[200\textrm{pA}, 400\textrm{pA}]$ and $[400\textrm{pA}, 800\textrm{pA}]$ for the interneuron cNAC 99111006, and only in the interval $[400\textrm{pA}, 600\textrm{pA}]$ for the interneuron cNAC 95817001. In order to construct an automatic rule (i.e. our Monod block procedure) based on condition (40), we proceed as follows. First, from the set of experimental spike times of a given neuron, we determine the range of currents $[I_{\textrm{block}}^{ I},I_{\textrm{block}}^{II}]$ for which condition (40) holds. Then, for each of them we calculate the straight line connecting the points $P_i = \left( I_{\textrm{stim}}, \, t \right)$, $i=1,2$, defined as

41 $$\begin{aligned} P_1=\left( I_{\textrm{block}}^{I}, \, t_{\textrm{spk}}^{\mathrm{\mathrm last}}(I_\textrm{block}^{I})+\frac{1}{2}ISI_{\mathrm{\mathrm last}}^{I}\right) , \quad 
P_2=\left( I_{\textrm{block}}^{II}, \, t_{\textrm{spk}}^{\mathrm{\mathrm last}}(I_\textrm{block}^{II})+\frac{1}{2}ISI_{\mathrm{\mathrm last}}^{II}\right) ,\nonumber \\ \end{aligned}$$

where $ISI_{\mathrm{\mathrm last}}^{I}=ISI_{\mathrm{\mathrm last}}(I_\textrm{block}^{I})$, and $ISI_{\mathrm{\mathrm last}}^{II}=ISI_{\mathrm{\mathrm last}}(I_\textrm{block}^{II})$. These two points can be considered as the extrema in $\left( I_{\textrm{stim}}, \, t\right)$-space of the firing dynamics. The choice of the prefactor 1/2 allows us to take into account potential small deviations between the model and the experimental values. Then, we consider the line joining $P_1$ and $P_2$ in $\left( I_{\textrm{stim}}, \, t\right)$-space, represented by the function

42 $$\begin{aligned} t= A_{I,II} I_{\textrm{stim}}+B_{I,II}, \end{aligned}$$

where

43 $$\begin{aligned} A_{I,II}= & {} \frac{\left( ISI_{\mathrm{\mathrm last}}^{II}+2 t_{\textrm{spk}}^\mathrm{\mathrm last}(I_{\textrm{block}}^{II})\right) -\left( ISI_{\mathrm{\mathrm last}}^{I}+2 t_{\textrm{spk}}^{\mathrm{\mathrm last}}(I_{\textrm{block}}^{I})\right) }{2 (I_\textrm{block}^{II}-I_{\textrm{block}}^{I})},\\ B_{I,II}= & {} \frac{I_{\textrm{block}}^{II} \left( ISI_{\mathrm{\mathrm last}}^{I}+2 t_{\textrm{spk}}^{\mathrm{\mathrm last}}(I_{\textrm{block}}^{I})\right) -I_\textrm{block}^{I} \left( ISI_{\mathrm{\mathrm last}}^{II}+2 t_{\textrm{spk}}^\mathrm{\mathrm last}(I_{\textrm{block}}^{II})\right) }{2 (I_{\textrm{block}}^{II}-I_\textrm{block}^{I})}. \nonumber \end{aligned}$$

Let $I_{\textrm{fire}}$ be the closest stimulation current above $I_\textrm{block}^{II}$ or below $I_{\textrm{block}}^{I}$ in the set $\left\{ 200\textrm{pA}, \, 400\textrm{pA}, \, 600\textrm{pA}, \, 800\textrm{pA}, \, 1000\textrm{pA} \right\}$ for which the firing block does not occur (as we show below, we have $I_{\textrm{fire}}=400$pA for the interneuron cNAC 99111006 and $I_{\textrm{fire}}=800$pA for the interneuron cNAC 95817001. Then the function (42) determines the time interval in which the Monod function (33) is defined according to the following rule

- for all $I_{\textrm{stim}}\le \displaystyle \frac{I_{\textrm{block}}^{II}+I_{\textrm{fire}}}{2}\equiv I_{\textrm{block}}^{\textrm{inf}}\;\;$ when $\;\;I_{\textrm{fire}}>I_{\textrm{block}}^{II}$;
- for all $I_{\textrm{stim}}\ge \displaystyle \frac{I_{\textrm{block}}^{I}+I_{\textrm{fire}}}{2}\equiv I_{\textrm{block}}^{\textrm{sup}}\;\;$ when $\;\;I_{\textrm{fire}}<I_{\textrm{block}}^{I}$.

The above procedure can be easily generalized when there is only one $I_{\textrm{block}}$ value that satisfies condition (40). In fact, in this case it is sufficient to set $I_{\textrm{block}}^{I}=I_{\textrm{block}}$, $I_{\textrm{block}}^{II}=I_{\textrm{fire}}$ when $I_{\textrm{fire}}>I_{\textrm{block}}$, and $I_{\textrm{block}}^{I}=I_\textrm{fire}$, $I_{\textrm{block}}^{II}=I_{\textrm{block}}$ if $I_{\textrm{fire}}<I_\textrm{block}$.

As an example, in the right panels of Fig. 3 we show how the Monod block procedure applies to the case of the interneurons cNAC 99111006 and 95817001.We recall that for the interneuron cNAC 99111006 condition (40) is satisfied for $I_\textrm{stim}=200,600,800$pA. Therefore, we have two block intervals given by

44 $$\begin{aligned} \begin{aligned} \left[ I_{\textrm{block}}^{I}, \, I_{\textrm{block}}^{II} \right] = \left[ 200\textrm{pA}, \, 400\textrm{pA} \right] , \quad \text { with } \quad I_{\textrm{fire}}=400\textrm{pA}, \\ \left[ I_{\textrm{block}}^{I}, \, I_{\textrm{block}}^{II} \right] =\left[ 600\textrm{pA}, \, 800\textrm{pA} \right] , \quad \text { with } \quad I_{\textrm{fire}}=400\textrm{pA}. \end{aligned} \end{aligned}$$

Moreover, the points $P_1$ and $P_2$ in these two cases are

$$\begin{aligned} \begin{aligned} P_1&=\left( 200\textrm{pA}, \, 259.95 \textrm{ms} \right) , \quad&P_2&=\left( 400\textrm{pA}, \, 396.10 \textrm{ms} \right) , \\ P_1&=\left( 600\textrm{pA}, \, 277.85 \textrm{ms} \right) , \quad&P_2&=\left( 800\textrm{pA}, \, 244.35 \textrm{ms} \right) , \end{aligned} \end{aligned}$$

respectively. Then, the functions (42) are

45 $$\begin{aligned} \begin{aligned}&t= 0.68\ I_{\textrm{stim}} + 123.80, \quad&\forall I_{\textrm{stim}}\le 300\mathrm pA,\\&t= - 0.17\ I_{\textrm{stim}}+378.35,\quad&\forall I_{\textrm{stim}}\ge 500\mathrm pA, \end{aligned} \end{aligned}$$

and they provide for each $I_{\textrm{stim}}$ the definition interval of the corresponding Monod function (33).

**Fig. 4:**
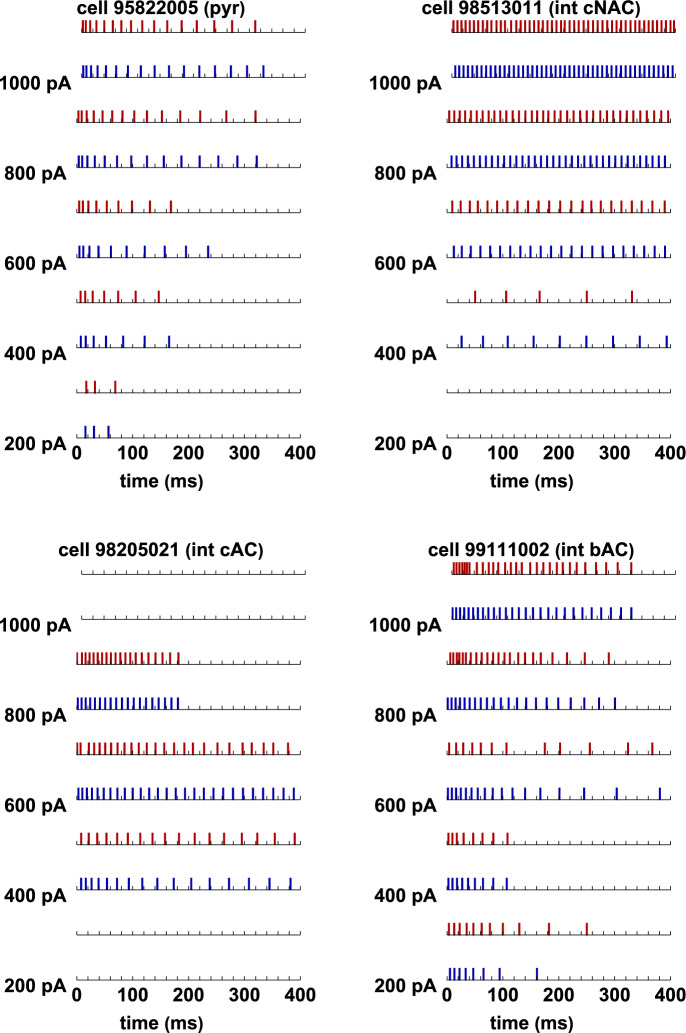
Typical optimization results. Raster plots representing spike times from experiments (red bars) and model (blue bars) for one pyramidal neuron and three interneurons exhibiting different firing patterns

**Fig. 5:**
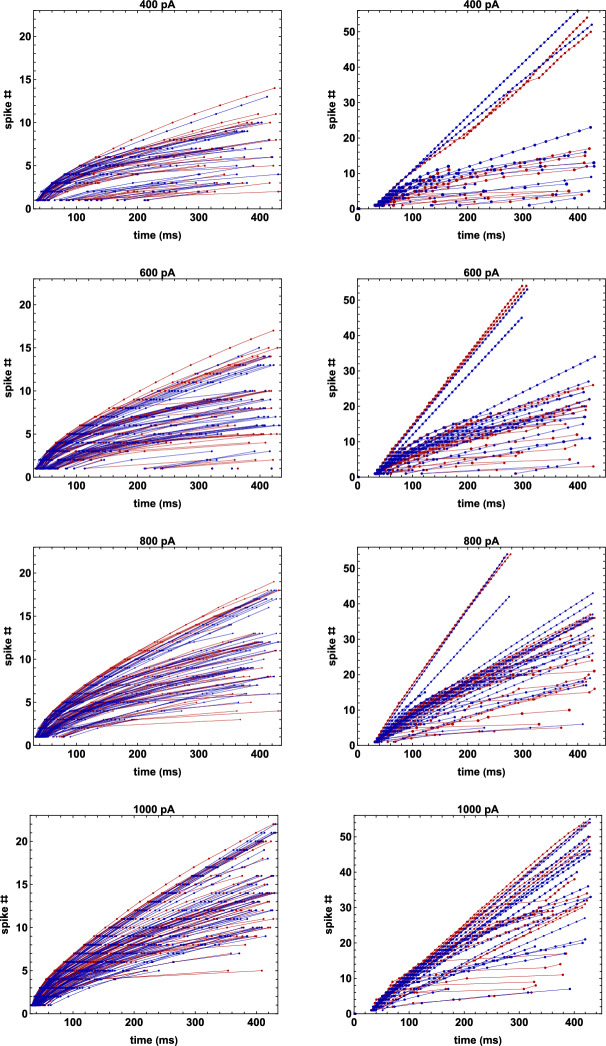
Comparison between experiments and models. Left: spike times of all pyramidal neurons considered in this work; (red experiments, blue model). Right: same as in the left panel but for interneurons (color figure online)

In the case of the interneuron cNAC 95817001 there is only one block interval given by

46 $$\begin{aligned} \left[ I_{\textrm{block}}^{I}, \, I_{\textrm{block}}^{II} \right] = \left[ 400\textrm{pA}, \, 600\textrm{pA} \right] , \quad \text { with } \quad I_{\textrm{fire}}=800\textrm{pA}. \end{aligned}$$

The points $P_1$ and $P_2$ read

$$\begin{aligned} p_1=\left( 200\textrm{pA}, \, 220.50 \textrm{ms} \right) , \quad P_2=\left( 400\textrm{pA}, \, 315.15 \textrm{ms} \right) , \end{aligned}$$

thus, we obtain

47 $$\begin{aligned} t=0.47\ I_{\textrm{stim}} + 31.20, \quad \forall I_{\textrm{stim}}\le 700 \mathrm pA. \end{aligned}$$

In the right panels of Fig. 3 we show the application of the Monod block procedure for the two interneurons cNAC 99111006 (upper panel) and cNAC 95817001 (lower panel). Note that the dashed portion of the curves indicates the time interval over which the neuron is expected to not fire according to the Monod block procedure, in agreement with the experimental recordings (see left panels in Fig. 3). These intervals are automatically calculated from experimental data, as shown above for the prototypical examples of two cNAC interneurons. We implemented the entire optimization workflow into a single python package (see model availability in Sect. 4), which reads the experimental data for all cells and carries out the parameter optimization and the Monod fitting with or without a block (see Suppl. Tables 3–5).

Typical examples of the optimization results are shown in Fig. 4, where we report the experimental raster plot for one pyramidal neuron and three interneurons. Note that the model cells were able to quantitatively reproduce not only the entire trains of spike times, but also the firing block in all cases. In Fig. 5 we show the full set of experimental spike times (Fig. 5, red markers and lines), compared with those obtained with the corresponding model cells (Fig. 5, blue markers and lines). As can be seen, the A-GLIF model framework was able to capture the full range of variability observed for both pyramidal neurons (Fig. 5A) and interneurons (Fig. 5B). In all cases, the model and experimentally observed spike times for all cells and all tested currents were statistically indistinguishable (see Suppl. Table 6 for p values).

**Fig. 6:**
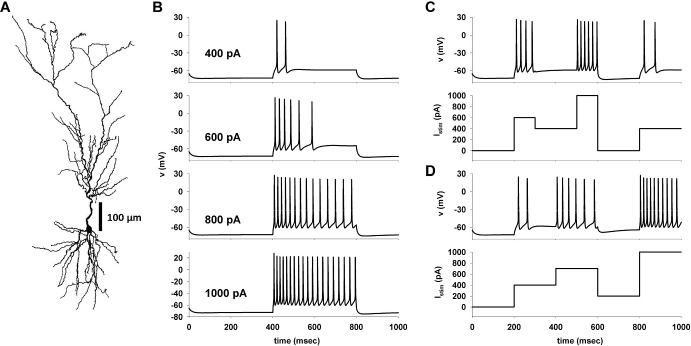
NEURON model. **a** The CA1 pyramidal neuron used for all simulations; **b** traces obtained for constant current injections; **c** traces obtained by a sequence of constant current injections; (**d**) same as in (**c**) but for a different current sequence

### Model validation

In this section, we test the ability of the A-GLIF implementation to also capture the spike time patterns observed under experimental protocols when applying current steps of different amplitudes not used to optimize the model. To this end, we adopted as a reference the traces generated by a realistic hippocampal CA1 pyramidal neuron model (Fig. 6a, see Sect. 4). The in-silico recordings under a constant current injection steps are shown in Fig. 6b, and they were similar to those experimentally recorded from real hippocampal CA1 pyramidal neurons. For the purpose of this paper, we also used recordings from simulations in which the neuron was stimulated with a series of current steps of different amplitude. Two representative cases are shown in Fig. 6c, d. It is important to note that the membrane voltage dynamics of a real neuron, during a period of constant current injection using a similar protocol, will be in general quite different from that observed during a constant current step at the same amplitude. An example can be seen in Fig. 6c during the two periods in which the current was held constant at 400 pA (300–500 ms, and 800–1000 ms). Under a constant 400 pA stimulation, the neuron should elicit two spikes (see top plot in Fig. 6b). However, in the first interval (Fig. 6c, 300–500 ms), following a 600 pA step, it did not generate any spike. The expected two spikes were instead generated during the second interval (Fig. 6c, 800–1000 ms), after a period without stimulation. However, this situation can be different for a different sequence, as shown in Fig. 6d for the two intervals at 600 pA (400–600 ms) and at 1000 pA (800–1000 ms). In this case, the firing pattern was very similar, although not identical, to what expected for constant injections (compare with the traces at 600 and 1000 pA in Fig. 6b). These behaviors are caused by the nonlinear dynamics of the ion channel currents, which can be captured by a biophysical accurate model but it is out of reach for any GLIF model calibrated only on constant currents. In the next section we will suggest further conditions that are able to reproduce this effect.

#### Constant stimulation current inside and outside the experimental range

**Fig. 7:**
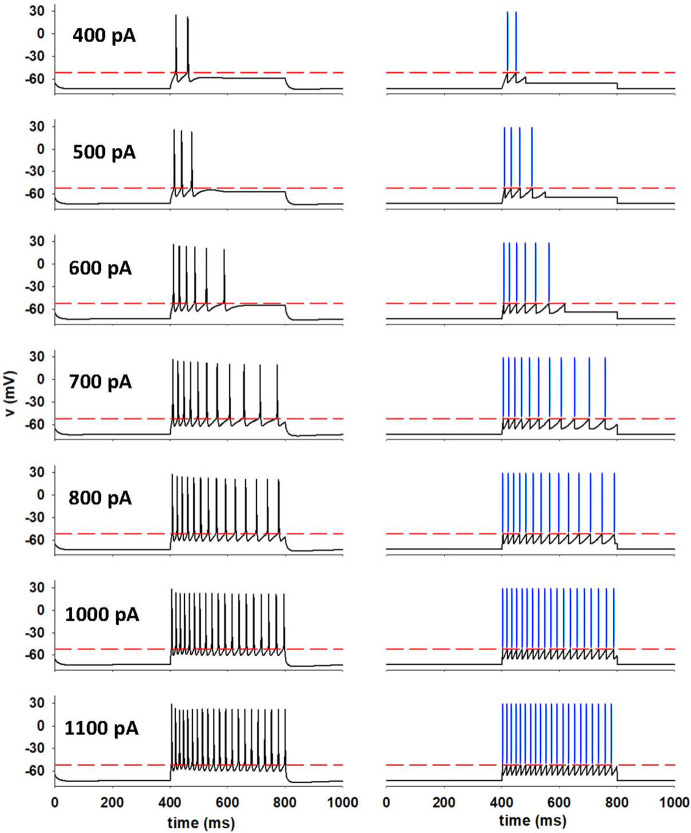
**Model validation for constant current injections.** Left: In-silico NEURON traces; red dashed lines represent $V_{th}$. Right: model traces; blue bars represent spike times (color figure online)

We optimized an A-GLIF model to reproduce the traces obtained with NEURON under a constant stimulation of 400, 600, 800, and 1000 pA. To validate the A-GLIF approach, we tested currents different from those used to optimize the model parameters, but still within the experimental range $\left[ I_{\textrm{stim}}^{\textrm{min}},I_{\textrm{stim}}^\textrm{max}\right]$ (i.e. 500 and 700 pA). All update rules and parameter constraints were automatically satisfied and no further changes and/or conditions were required to quantitatively reproduce the experimental traces, as shown in Fig. 7 where the experimental traces (Fig. 7, left plots) are compared with those obtained with the A-GLIF model (Fig. 7, right plots).

We then focused our attention on constant stimulations outside the experimental range, distinguishing two cases: *(1)* positive currents beyond the experimental range and *(2)* positive or negative currents below $I_{\textrm{th}}$, which need additional considerations as explained below.

*(1)*: If we consider a positive stimulation $I_\textrm{stim}$ outside the range experimentally tested, i.e. $I_\textrm{th}<I_{\textrm{stim}}<I_{\textrm{stim}}^{\textrm{min}}$ or $I_{\textrm{stim}}>I_\textrm{stim}^{\textrm{max}}$, the sequence of initial data $I_{\textrm{adap}}^0 (\chi , I_{\textrm{stim}})$ in Eq. (33) may not satisfy the positivity condition in Eq. (35) and/or the threshold condition provided in Eq. (37). We observe that in the optimization procedure it is required that the threshold condition (37) is satisfied for all $I_{\textrm{stim}} \in \left[ I_\textrm{stim}^{\textrm{min}}, I_{\textrm{stim}}^{\textrm{max}} \right]$ at least in the interval $\left[ t_{\textrm{spk}}^{\textrm{first}}\left( I_\textrm{stim}\right) -t_{\textrm{start}},t_{\textrm{spk}}^{\mathrm{last-1}}\left( I_\textrm{stim}\right) -t_{\textrm{start}} \right]$ with $t_{\textrm{spk}}^\mathrm{last-1}\left( I_{\textrm{stim}}\right) -t_{\textrm{start}} < T$ and $t_\textrm{spk}^{\mathrm{last-1}}\left( I_{\textrm{stim}}\right) -t_{\textrm{start}}$ eventually defined through the Monod block procedure described in Sect. 2.4. Nevertheless, for $I_{\textrm{stim}} \in \left[ I_{\textrm{stim}}^{\textrm{min}}, I_{\textrm{stim}}^{\textrm{max}} \right]$, Eq. (37) may not be satisfied for all times; imposing that Eq. (37) holds for all $I_{\textrm{stim}} > I_{\textrm{th}}$ outside the interval $\left[ I_{\textrm{stim}}^{\textrm{min}}, I_{\textrm{stim}}^{\textrm{max}} \right]$ is hence quite restrictive. In our numerical procedure, we will thus prioritize the positivity property of the Monod function given in Eq. (35) over the threshold condition (37); this might hence lead to an initial decrease in the potential *V* after a spike event has occurred. We report the conditions which ensure the validity of Eqs. (35) and (37) outside the range $\left[ I_{\textrm{stim}}^{\textrm{min}}, I_{\textrm{stim}}^{\textrm{max}} \right]$ in the case $a > 0$, as this is the most common situation found in this work (84 CA1 neurons, the computational NEURON model, and the Layer 5 visual cortical neuron). The full analysis, also including the other scenarios, is carried out in Suppl. Sec. 4.4. In this case, when the Monod function is negative, to satisfy the positivity condition (35) and, eventually, also the threshold condition (37) it is sufficient to translate the Monod function along the vertical axis. One possibility which would ensure the validity of Eq. (37) consists in considering
  48 $$\begin{aligned} \left( I_{\textrm{adap}}^0(\chi ,I_{\textrm{stim}})\right) ^*:=c^{*}+\frac{a\exp (b \, I_{\textrm{stim}})\chi }{d+\chi }, \end{aligned}$$
  where
  49 $$\begin{aligned} c^{*}:=\frac{\alpha }{\beta }+\eta I_{dep}^{0}+\frac{\delta }{\beta } \left( 1+V^{0}\right) -a\exp (b \, I_{\textrm{stim}}),\quad \eta \in \left[ 0,1\right] , \end{aligned}$$
  as long as $\left( I_{\textrm{adap}}^0(\chi ,I_{\textrm{stim}})\right) ^*$ satisfies the positivity condition (35). The value of $\eta$ in Eq. (49) can be chosen in order to minimize the distance from the original Monod function $I_{\textrm{adap}}^0(\chi ,I_\textrm{stim})$. If the choice of $c^*$ in (49) does not ensure the positivity of the modified Monod function, an alternative possibility is to consider
  50 $$\begin{aligned} c^*=c-L, \end{aligned}$$
  where $L=I_{\textrm{adap}}^0(t_{\textrm{spk}}^{\textrm{first}}\left( I_\textrm{stim}\right) ,I_{\textrm{stim}})<0$. This ensures the positivity of the Monod function for the interval over which spikes occur, i.e. our range of interest.
*(2)*: If $0<I_{\textrm{stim}}<I_{\textrm{th}}$, there are no spikes and the system will then reach an equilibrium configuration $E_1$, in which the membrane potential tends to $V_1^*=\alpha /(\beta -\delta )-1$. If $I_{\mathrm{{stim}}}<0$, the membrane potential must relax to a value below $E_{\textrm{L}}$. To limit the minimum voltage to the physiologically plausible value of $V_\textrm{min}=-90$mV (corresponding to the reversal potential of potassium currents), we have chosen to set the equilibrium value as follows:
  51 $$\begin{aligned} \begin{aligned} V_1^*&= -1+\frac{(1+V_{\textrm{min}}^*)\alpha }{\alpha ^{\textrm{neg}}}, \quad \forall I_{\textrm{stim}}: I_{\textrm{stim}}^{\textrm{neg}}\le I_{\textrm{stim}} \le 0, \\ V_1^*&= V_{\textrm{min}}^*, \quad \forall I_{\textrm{stim}}: I_{\textrm{stim}} < I_{\textrm{stim}}^{\textrm{neg}}, \end{aligned} \end{aligned}$$
  where $\alpha ^{\textrm{neg}}=I_{\textrm{stim}}^{\textrm{neg}}/K$, $V_\textrm{min}^*=-V_{\textrm{min}}/E_{\textrm{L}}$, and $I_{\textrm{stim}}^{\textrm{neg}}$ is the, experimentally measured, current for which the cell relaxes to $V_{\textrm{min}}=-90$mV. For our NEURON cell, $I_{\textrm{stim}}^\textrm{neg}=-185$ pA.

With these additional conditions, the model is now able to reproduce any constant stimulation protocol. In Fig. 7 we compare the NEURON traces (Fig. 7, left) with those obtained via the A-GLIF model (Fig. 7, right) for a series of constant current stimulations, including amplitudes that were not used for the optimization (i.e. 500, 700, and 1100 pA).

#### Piecewise constant stimulation currents

We now consider the case in which the injected current $I_\textrm{stim}(t)$ is a constant piecewise continuous function on the interval $[T_0,T_N]$, i.e., it is a function defined and continuous on this interval except for a finite number of discontinuities.

In particular, we assume that in the time intervals $$[T_0,T_1),[T_1,T_2),...,[T_{N-1},T_N]$$ the neuron is stimulated by the constant currents $$I_1,I_2,...,I_N$$, respectively. It should be evident that our model can be applied in each of the above time intervals, and in each of them all the qualitative and quantitative results on the equilibria, solutions, and parameter constraints still hold.

To take into account the discontinuities in the injected current, the model need to be equipped with additional initial conditions, to take into account the current change at time instants ${\bar{t}}=T_1,...,T_{N-1}$.

- Let ${\bar{t}}$ be a time such that $I_{\textrm{th}}<I_\textrm{stim}({\bar{t}})<I_{\textrm{stim}}({\bar{t}}-\Delta t)$. From a physiological point of view, the only constraint for the model after such a change in the current is that the membrane voltage should still be increasing, i.e. ${\dot{V}}({\bar{t}})>0$. Considering Eq. (10)$_1$ and Eq. (30), this condition can be obtained by setting *V*, $I_{\textrm{dep}}$, and $I_\textrm{adap}$ as follows
  52 $$\begin{aligned} V^0({\bar{t}})=V({\bar{t}}),\quad I_{\textrm{adap}}^0({\bar{t}})=I_\textrm{adap}({\bar{t}}), \quad I_{\textrm{dep}}^0({\bar{t}})=I_\textrm{adap}^0({\bar{t}})+\Theta ^\star \frac{{\tilde{\alpha }}}{\beta }, \end{aligned}$$
  where ${\bar{\alpha }} =I_{\textrm{stim}} ({\bar{t}})/K$ and the constant $\Theta ^\star$ is defined as
  53 $$\begin{aligned} \Theta ^\star = {\left\{ \begin{array}{ll} \Theta &{} \text{ if }\ \Theta \ge 0,\\ 0 &{} \text{ if }\ \Theta <0, \end{array}\right. } \end{aligned}$$
  where
  54 $$\begin{aligned} \Theta =\frac{V({\bar{t}}-\Delta t)}{\bar{{\bar{\alpha }}}}\left( \frac{1}{\Delta t}-\delta \right) -\frac{V({\bar{t}}-2\Delta t)}{\bar{{\bar{\alpha }}}\Delta t}-\frac{\delta }{\bar{{\bar{\alpha }}}}-1, \end{aligned}$$
  and $\bar{{\bar{\alpha }}} =I_{\textrm{stim}} ({\bar{t}}-\Delta t)/K$. This allows us to choose the same (non negative) value of theta as for ${\bar{t}}-\Delta t$, i.e.,
  $$\begin{aligned} I_{\textrm{dep}}\left( {\bar{t}}-\Delta t\right) =I_{\textrm{adap}}\left( {\bar{t}}-\Delta t\right) +\Theta \frac{\alpha \left( {\bar{t}}-\Delta t\right) }{\beta }. \end{aligned}$$
- If the current increases at ${\bar{t}}$, i.e. $I_\textrm{stim}({\bar{t}})>0$ and $I_{\textrm{stim}}({\bar{t}})>I_\textrm{stim}({\bar{t}}-\Delta t)$, then the system just continues with its dynamics, with initial conditions thus updated as
  55 $$\begin{aligned} V^0({\bar{t}})=V({\bar{t}}), \quad I_{\textrm{adap}}^0({\bar{t}})=I_\textrm{adap}({\bar{t}}), \quad I_{\textrm{dep}}^0({\bar{t}})=I_{\textrm{dep}}({\bar{t}}). \end{aligned}$$

It should be noted that the dynamical behavior of the membrane potential in the presence of piecewise constant currents is, in general, rather different from what can be observed for constant stimulation currents (see Fig. 6).

**Fig. 8:**
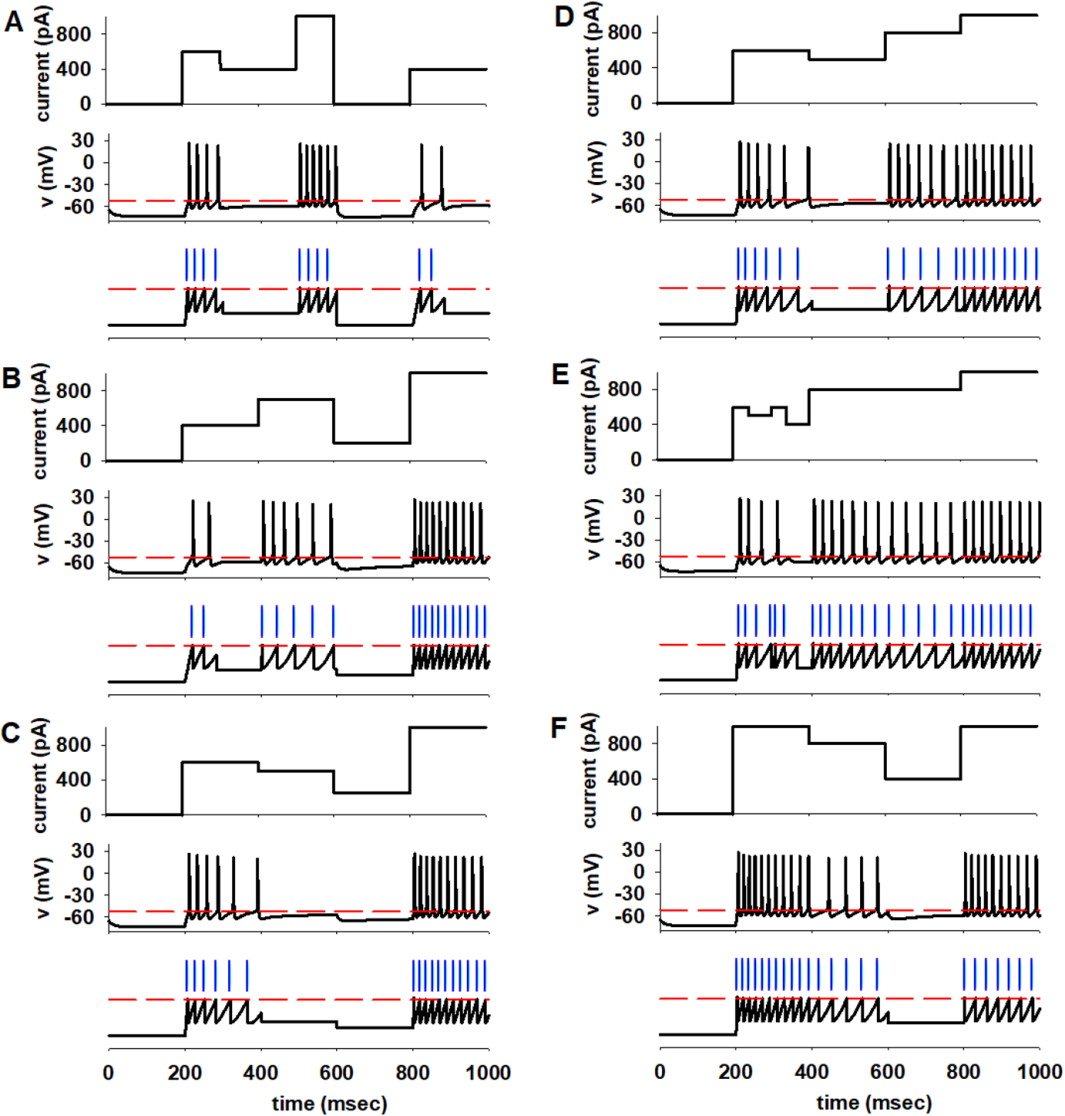
**Model validation for piecewise currents.** **a** (top) current steps during a 1 sec long simulation; (middle) experimental NEURON trace (red dashed line represents $V_{th}$); (bottom) model traces, blue bars represent spike times. (**b**–**f**) as in A but with a different current sequence (color figure online)

Several different combinations of stimulation currents are reported in the top plots in Fig. 8 (panels a–f). As can be seen from the middle plots in all panels if the stimulation current decreases, the neuron generally stops firing (e.g. see Fig. 8a, between 300–500 ms) instead of eliciting spikes (see Fig. 7 for 400pA); if the current increases, the neuron may still fire, but the number of spikes is lower than those expected (e.g. 11 spikes during the first 200 ms under a constant current injection from rest, as in Fig. 7, instead of the 10 spikes generated after a 200pA step, 800–1000ms in Fig. 8d). To reproduce this behaviour, it is necessary to update the initial conditions for $I_{\textrm{adap}}$ after each current change. We found that when $I_{\textrm{stim}}(t)\ge I_\textrm{stim}(t-\Delta t)\ge I_{\textrm{th}}$, the sequence of $I_{\textrm{adap}}^0$ can still be obtained from the Monod function (33) provided that the value of $t_{\textrm{start}}$ is updated as follows

56 $$\begin{aligned} t_{\textrm{start}}\quad \rightarrow \quad t_{\textrm{start}}\left( 1+\frac{I_\textrm{stim}(t-\Delta t)-I_{\textrm{th}}}{I_{\textrm{stim}}(t-\Delta t)}\right) . \end{aligned}$$

With these update rules we obtain a model that is able to quantitatively reproduce any piecewise constant stimulation, as shown in the bottom plots of all panels in Fig. 8 (see Suppl. Table 2).

### Model validation using different current protocols and comparison with a state of the art model

**Fig. 9:**
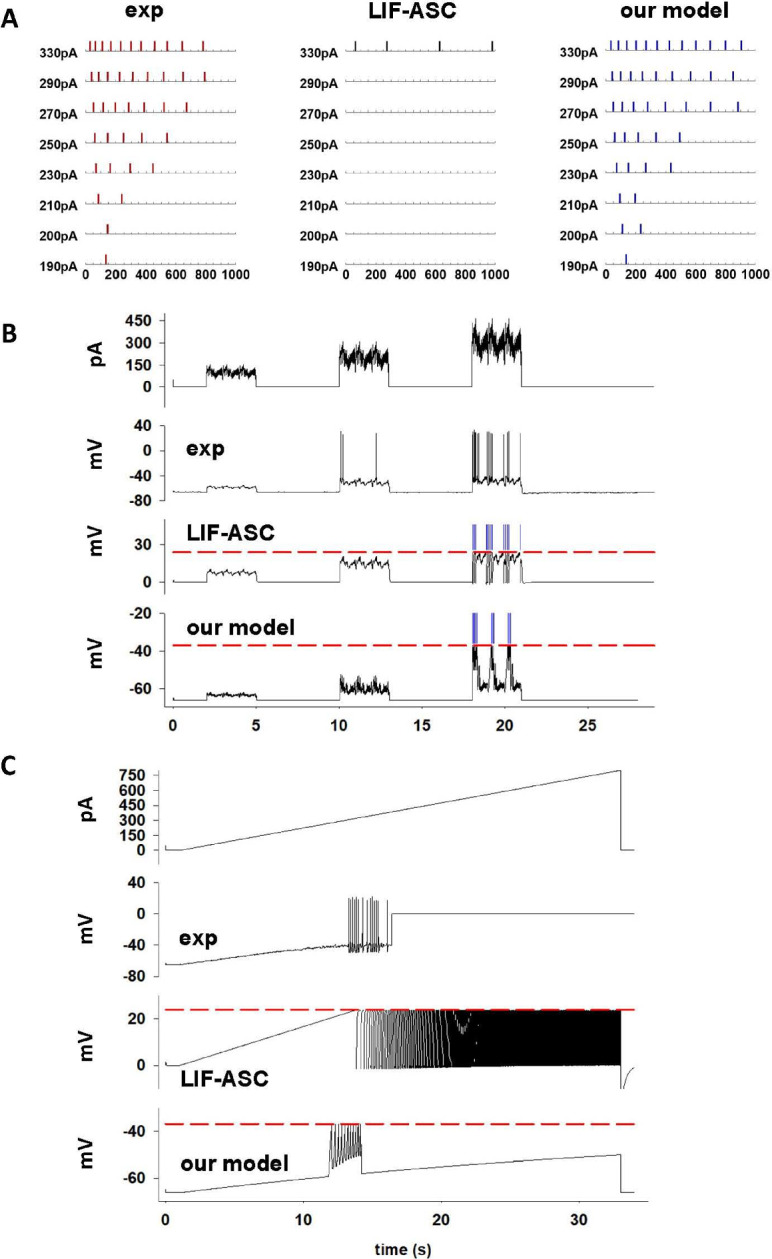
Model’s performance under different current stimulation protocols. **a** Constant current injection; (left) experimental spike times, (middle) spike times obtained with the LIF-ASC model, (right) spike times from our model. **b** Response of the same neuron in (**a**) to a dynamic current clamp protocol; the top plot represents the experimental current, the other plots are the response obtained from a real neuron, the LIF-ASC model, and our model; blue lines in the plots for the LIF-ASC and our model represent spike times. (**c**): Response to a ramp current; the top plot represents the experimental current injected into the same neuron in (**a**) and (**b**), the other plots represent the response recorded from the neuron, the LIF-ASC model, and our model. All results for the LIF-ASC model are taken from Allen (2018) (color figure online)

In order to validate the model using different experimental current injection protocols, we adopted as a reference a set of experimental traces recorded from an excitatory Layer 5 visual cortical neuron (cell id 476048909, from Allen (2018)) in response to different somatic current injection protocols (see Teeter et al (2018)): (1) constant steps, (2) dynamic clamp, generated with pink noise stimuli (3 s each, 1/f distribution of power, 1–100 Hz) with amplitudes centered at 75, 100, and 125 percent of the neuron rheobase, (3) a ramp, i.e. increasing amplitude at a rate much slower than the time constant of the neuron. In all cases, we compared the results obtained with our model with those obtained with the modeling approach presented in Teeter et al (2018), and in particular with the LIF Afterspike Currents (LIF-ASC) model. For this purpose, we first created an A-GLIF version of cell 476048909, by applying our optimization workflow for constant current injections (all model parameters are reported in Suppl. Table 3). The results are presented in Fig. 9a. We obtained, also in this case, a very good agreement with experimental findings, in contrast with the LIF-ASC model, which failed essentially over the entire range of currents. We then validated our model against the experimental spike times obtained in response to the dynamic clamp current (Fig. 9b). In this case, the LIF-ASC model produced very good results, in comparison with those obtained with our model. This was expected since the LIF-ASC model was optimized also using these traces. Finally, our model was in very good agreement with the recording obtained applying the ramp protocol (Fig. 9c), again in striking contrast with the LIF-ASC model’s results. These results demonstrate the ability of the A-GLIF model to be consistent with experimental findings obtained under experimental protocols substantially different from those used for the optimization procedure.

## Discussion

The implementation of simplified neuron models, able to accurately capture the experimentally observed spike times, is of paramount importance to mitigate the technical limitations of current supercomputing systems in running large-scale models of brain regions at single cell resolution.

As pointed out in Gerstner and Naud (2009), an optimal neuron model should consider tuning parameters on a neuron-by-neuron basis, should include adaptation, and its quality should be measured on data not used during the tuning phase. Following these indications, in this paper we have introduced a mathematical framework, built upon the GLIF approach, which is able to reproduce all the main firing properties of individual hippocampal CA1 pyramidal neurons (including adaptation and bursting), and validated against recordings not used to optimize the parameters; we termed it A-GLIF. The rationale for introducing the A-GLIF framework, is that the original LIF equations are linear and with fixed reset rules, making it impossible to reproduce the Hodgkin-Huxley type of nonlinear dynamics exhibited by real neurons. The main goal of this paper was to maintain the linear nature of the equations, since they lead to extremely useful analytic solutions, and introduce a new set of conditions and update rules capturing the nonlinear nature of CA1 neuron dynamics.

A couple of notable attempts to deal with this problem are the GLIF approach of Geminiani et al (2018), which captured the complex spiking behaviour of cerebellar neurons, and that of Teeter et al (2018), which reproduced the experimental dynamics observed under dynamic clamp protocols. However, the condition imposed on the model parameters in Geminiani et al (2018) constrains the threshold potential to be a stable equilibrium, whereas to capture the CA1 neuron dynamics, an asymptotically stable equilibrium is needed. In Teeter et al (2018), the optimization procedure used experimental protocols that are not routinely carried out in laboratories, and the resulting models are not able to reproduce at the same time experimental findings with constant or variable current injections.

There are two main features making this model different, and more accurate, with respect to any other similar implementation based on a leaky or an adaptive exponential integrate-and-fire scheme: (1) a compact representation of the general analytical solutions using only three parameters, leading to (i) exact trajectories that outperform in terms of speed and accuracy any other available optimization procedure, and (ii) analytical conditions to reproduce both constant and variable current injections; (2) the use of a mathematically derived set of constraints on the update rules after a spike, which are able to capture the main experimentally observed firing properties, including those that cannot be reproduced by any other published GLIF approach.

These results may have a significant (positive) impact on the implementation of large-scale network models. The equilibrium and stability analysis of the analytical solutions, allowed to find constraints on the parameter space that can drastically reduce the computational resources needed to optimize model parameters. Nevertheless, implementation speed and trajectory accuracy are not the only advantages of the approach discussed in this work. One of the main results is that, by analyzing the experimental firing properties, we were able to find a scheme to quantitatively characterize and predict, through a Monod function, the firing behaviours of hippocampal pyramidal neurons and interneurons, in response to any stimulation protocol using piecewise constant current injections. This solves a serious limitation of all large scale networks implementation based on spiking neurons, namely the use of identical copies for the cells composing the network. Our approach allows to easily generate an arbitrary number of statistically representative neurons with different firing properties and spike times, but still within the experimentally observed range (Marasco et al 2023). It would thus be rather straightforward to reproduce the physiological variability, with a significant (positive) impact on the implementation of large-scale network models.

One limitation of literally all models based on the linear LIF equations is that they cannot quantitatively reproduce the nonlinear behavior of a neuron under a time-varying current. This well known limitation is accepted by the community, in return for a great reduction in computational requirements, mathematical tractability and, at this time, as being the only possibility to implement full-scale models of entire brain regions. In this latter case, additional tuning must be carried out to adjust the synaptic transmission properties to reproduce specific network behaviors directly observed or inferred from specific experimental findings. In the approach presented here, we used current steps to help other researchers to implement their models using classic *in vitro* recordings, where constant current steps of different amplitude and duration are universally used in the field to assess firing behaviors and neuron excitability properties. More complex experimental protocols to generate electrophysiological traces (such as dynamic clamp, ramps, zaps, etc.) are much less common and are not a better representation of natural conditions, where an unknown number of synaptic inputs targets unknown dendritic locations on an unknown morphology. Unfortunately, under these conditions, it is practically impossible to record traces under evoked synaptic inputs *in vitro* or *in vivo* that can be directly used to optimize a model. Although synaptic currents could be considered as an extreme case of piecewise constant inputs, since they change at each time step, their comprehensive consideration requires a more extensive and detailed investigation. In conclusion, the framework presented in this work is an important step toward a single cell model implementation able to accurately reproduce the excitability properties of hippocampal neurons and interneurons. The model, as is, was also able to be in qualitative agreement with experimental recordings under a protocol mimicking synaptic inputs. In a future work, we will introduce and discuss a suitable set of update rules taking into account also a large set of synaptic inputs.

## Materials and Methods

### Experimental Data Used for Modeling

**Fig. 10:**
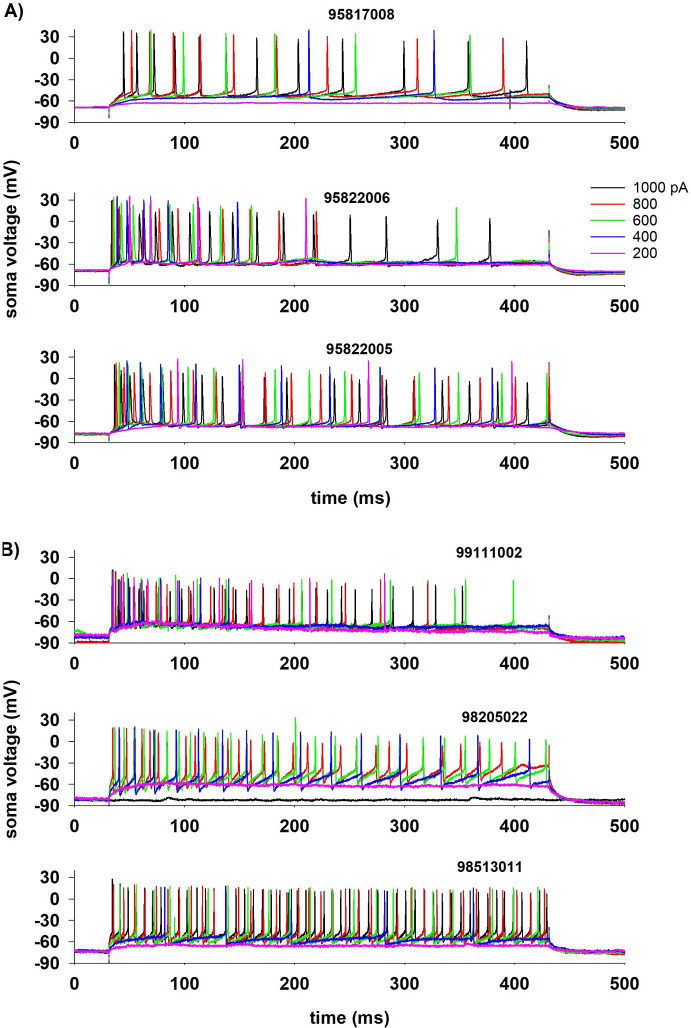
Reference experimental traces. **a**: Typical somatic recordings from three CA1 pyramidal neurons. **b**: Typical somatic recordings from CA1 interneurons

**Fig. 11:**
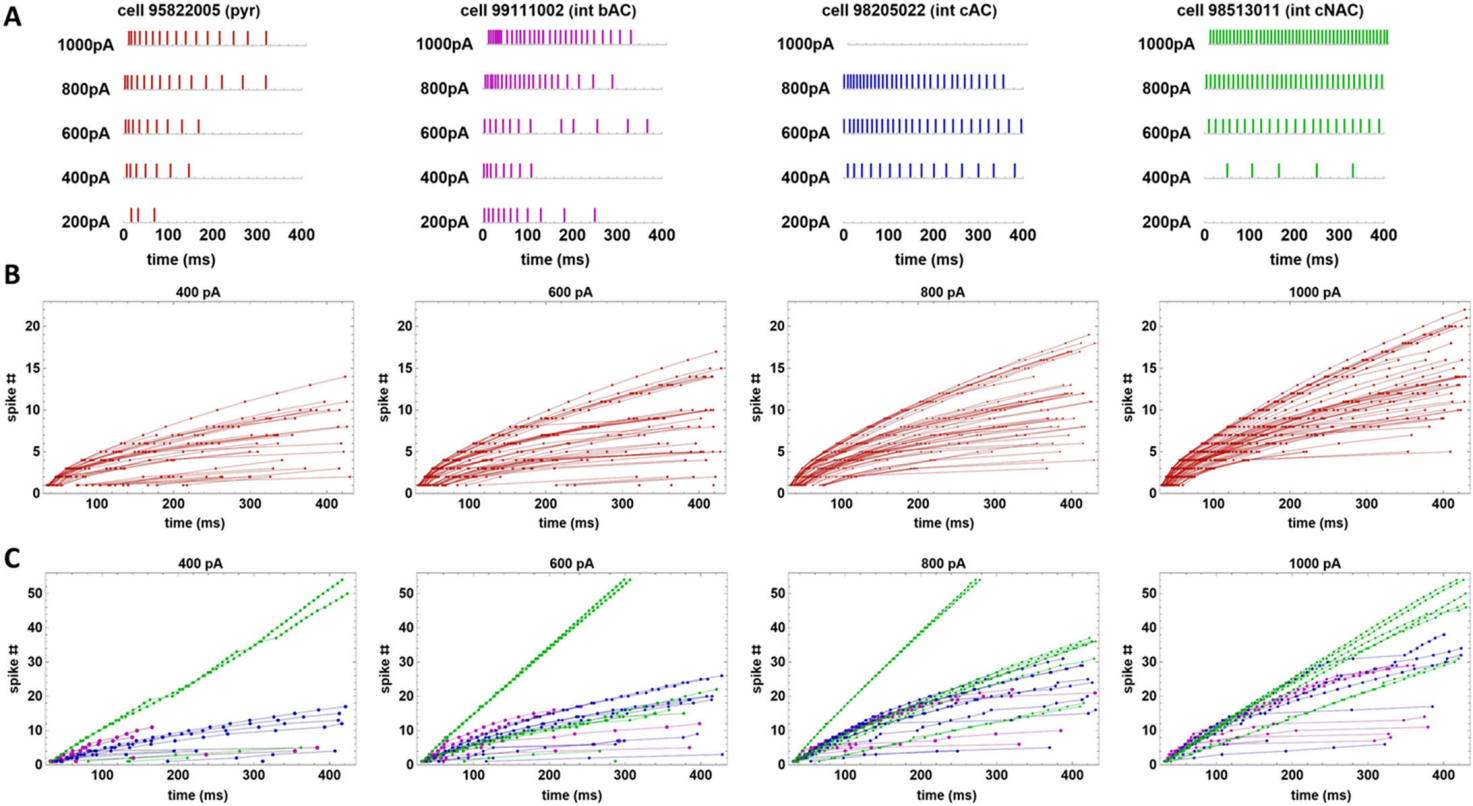
Reference experimental data. **a** Typical raster plots from one pyramidal neuron and three interneurons, representing the different firing behaviour observed experimentally. **b** spike number as a function of the spike times for all the pyramidal neurons considered in this work. **c** as in B but for CA1 interneurons. Marker and line colors represent different firing properties

To test and validate our model we considered a set of somatic voltage traces recorded from 84 cells: 58 pyramidal and 26 interneurons, obtained from *in vitro* rat hippocampal CA1 slices (Migliore et al 2018), in response to somatic constant current injections, from $I_{\textrm{stim}}^{\textrm{min}}=$200pA to $I_\textrm{stim}^{\textrm{max}}=$1000pA with a step of 200pA. The 314 traces from pyramidal neurons were all classified as *continuous accommodating cells* (cAC); for interneurons, 54 traces were classified as cAC, 72 traces as *bursting cells* (bAC), and 62 traces as *continuous non-accommodating cells* (cNAC). Typical examples illustrating the physiological variability observed for these classification are shown in Fig. 10. Note the large variability, in response to the same input, observed for both pyramidal cells (Fig. 10a) and interneuron (Fig. 10b). This is particularly evident at low currents. In this work we were interested in reproducing the spike times. Typical raster plots for both type of cells are shown in Fig. 11a, and the full set of spike times for all currents investigated experimentally are plotted in Fig. 11b for pyramidal neurons and in Fig. 11c for interneurons.

### Single cell NEURON simulations used for modeling

For a set of specific current injection protocols, which were not available experimentally and that we wanted to use as a reference for model validation, we considered a NEURON (Hines and Carnevale (1997),v8.0.0) model. For this purpose, a realistic morphological and biophysical reconstruction of a hippocampal pyramidal CA1 neuron (cell oh140807_A0_idB from Migliore et al (2018), see Fig. 6a), was adapted to generate the specific cAC firing patterns illustrated in Fig. 6B. We tested both constant (Fig. 7), and piecewise constant current injections (Fig. 8). To avoid confusion with the A-GLIF model traces, we will always refer to the NEURON model results as “experimental traces”.

All model and simulation files are available in the ModelDB section of the Senselab database at the link http://modeldb.yale.edu/267598 and in the EBRAINS Live Papers collection (Appukuttan et. al 2023) at the link https://live-papers.brainsimulation.eu/.

### Optimization procedure

A custom procedure for parameter optimization was carried out using the *geneticalgorithm()* python library, with 200 individuals and a maximum of 250 generations. The procedure stops when the error between two consecutive generations does not improve by more than 2%.

To determine the A-GLIF models for the 84 cells and the NEURON model, we first extracted from the somatic traces the resting potential $E_{\textrm{L}}$, the reset potential $V_r$, and the threshold potential $V_{\textrm{th}}$, in addition to all spike times at all currents. Although, in general, parameters like the rheobase current $I_{\textrm{th}}$, the membrane capacitance $C_{\textrm{m}}$, and the membrane time constant $\tau _{\textrm{m}}$, can also be inferred from the experimental traces, or fixed according to the literature, we have preferred to treat them as fitting parameters, together with all the other model parameters, $K, k_{\textrm{adap}}$ and the initial conditions for $I_{\textrm{dep}}$ and $I_{\textrm{adap}}$. Their final values after the optimizing procedure for each cell are reported in Table S1, together with all other parameters extracted from the traces. It is important to note that the use of the analytical solution allowed a full vectorization (and thus parallelization) of the optimization procedure, in contrast to the computationally expensive simulation runs with the ODEs that would have been needed to evaluate a cost function. In our case, we decided to focus the cost function on the time of first spike and to the ISI sequences simultaneously for all tested currents, and it was thus defined as

57 $$\begin{aligned} \begin{aligned} \mathrm{cost_{func}}=&\sum _{i=1}^{N_c}\Vert t_{\textrm{spk M}(i)}^\textrm{first}-t_{\textrm{spk E}(i)}^\textrm{first}\Vert +\sum _{i=1}^{N_c}\sum _{j(i)=1}^{n_i-1} \max \left( 0, ISI^{\textrm{exp}}_{j(i)}-ISI^{\textrm{max}}_i\right) \\&+\sum _{i=1}^{N_c}\sum _{j(i)=1}^{n_i-1} \max \left( 0, ISI^\textrm{min}_i-ISI^{\textrm{exp}}_{j(i)}\right) , \end{aligned} \end{aligned}$$

where $I_1,...,I_{N_c}$ are the injected constant stimulation currents, $N_c \le 5$, and $n_i$ the number of the somatic spikes generated by the $i-$th stimulation current $I_i$, $t_{\textrm{spk M}(i)}^{\textrm{first}},t_{\textrm{spk E}(i)}^{\textrm{first}}$ are the model and experimental first spike-time for the injected current $I_i$, respectively. Moreover, $ISI^{\textrm{exp}}_{j(i)}$ denotes the $j-$th experimental ISI obtained in response to the stimulation current $I_i$, and $ISI^{\textrm{max}}_i, ISI^{\textrm{min}}_i$ are, respectively, the maximum and minimum values of the expected ISI for the $i-$th stimulation current. We remark that in view of Eqs. (30) and (31) the maximum and minimum values of the model ISIs are obtained by setting the values of $I_{\textrm{adap}}^0$, respectively, as follows

58 $$\begin{aligned} I_{\textrm{adap}}^0 = \frac{\alpha _i}{\beta }+I_\textrm{dep}^0+\frac{\delta }{\beta } (1+V^0),\qquad I_{\textrm{adap}}^0 =0, \end{aligned}$$

where $$\alpha _i=I_i/K$$.

For each stimulation current $I_i$, the optimization procedure provided a sequence of data

59 $$\begin{aligned} \left( t_{\textrm{spk}(1)}^+, I_{\textrm{adap}}^0(t_{\textrm{spk}(1)}^+, I_i)\right) ,...,\left( t_{\textrm{spk}(n_i-1)}^+, I_{\textrm{adap}}^0(t_{\textrm{spk}(n_i-1)}^+, I_i)\right) \end{aligned}$$

that were fitted by the Monod-type function (33) taking into account the parameter constraints defined in items *(i)* and *(ii)* of Sect. 2.4.^3^

For this purpose, we adopted the fitting method described in Gao and Lixing (2012) to obtain the coefficients *a*, *b*, *c*, *d* of Eq. (33) by minimizing the following cost function

60 $$\begin{aligned} \textrm{cost}_{\textrm{Monod}}=\sum _{i=1}^{N_c}\sum _{j(i)=1}^{n_i-1}\left( c+\frac{a \, e^{b \, I_i} \, (t_{\textrm{spk}j(i)}^+-t_{\textrm{start}})}{d +(t_{\textrm{spk}j(i)}^+-t_{\textrm{start}})}-I_{\textrm{adap}}^0(t_{\textrm{spk}j(i)}^+, I_i)\right) ^2. \end{aligned}$$

In those cases in which we did not obtained a good fit, we used the built-in function *NonlinearModelFit* of the software Mathematica (ver. 13.01, Wolfram), which implements fitting algorithms based on conjugate gradient, gradient, Levenberg–Marquardt, Newton, NMinimize, and quasi-Newton methods.

The optimization and the python simulation code, together with a NEST implementation of the A-GLIF model using the analytical solutions, will be available in the ModelDB section of the Senselab database (http://modeldb.yale.edu/267598), and in the live papers section of EBRAINS (https://live-papers.brainsimulation.eu/).

### Statistical Analysis

To statistically verify that the A-GLIF model was able to reproduce the spike trains in response to constant and piecewise constant stimulation currents we relied on the *Mann–Whitney U-test* using the built-in function *MannWhitneyTest* of the software Mathematica (ver. 13.01, Wolfram). For each of the 84 hippocampal CA1 pyramidal neurons and interneurons, and for the NEURON and the Layer 5 visual cortical neuron models we considered the paired samples $\mathrm{data_{\textrm{exp}}}$ (experimental data) and $\mathrm{data_{\textrm{mod}}}$ (model data) of length *n* and *m*, respectively, as follows

61 $$\begin{aligned} \begin{aligned}&\mathrm{data_{\textrm{exp}}}=\left\{ (I_{\textrm{stim}}^{1}, {\bar{t}}_\textrm{spk}^1),...,(I_{\textrm{stim}}^{1}, {\bar{t}}_{\textrm{spk}}^{n_1}),....,(I_\textrm{stim}^{N}, {\bar{t}}_{\textrm{spk}}^1),...,(I_{\textrm{stim}}^{N}, {\bar{t}}_\textrm{spk}^{n_N})\right\} ,\; \left( n=\sum _{i=1}^{N} n_i\right) , \\&\mathrm{data_{\textrm{mod}}}=\left\{ (I_{\textrm{stim}}^{1}, t_\textrm{spk}^1),...,(I_{\textrm{stim}}^{1}, t_{\textrm{spk}}^{m_1}),....,(I_\textrm{stim}^{N}, t_{\textrm{spk}}^1),...,(I_{\textrm{stim}}^{N}, t_\textrm{spk}^{m_N})\right\} , \; \left( m=\sum _{i=1}^{N} m_i\right) , \end{aligned}\nonumber \\ \end{aligned}$$

where $n_h$ and $m_h$ are the number of the experimental ${\bar{t}}_{\textrm{spk}}^i$ and modeled $t_{\textrm{spk}}^j$ spike times, respectively, in the $h-$th trace relative to the constant stimulation current $I_{\textrm{stim}}^{h}$.

After having verified that the data were elliptically symmetric, we tested whether the median of bivariate sample $\mathrm{data_{\textrm{exp}}}$ and $\mathrm{data_{\textrm{mod}}}$ were equal performing an extension of the Mann-Whitney U-test using the spatial ranks. In particular, we tested the null hypothesis $H_0: \mu _{1}=\mu _{2}$ against the alternative hypothesis $H_a: \mu _{1} \ne \mu _2$, where $\mu _{1}$ and $\mu _{2}$ are the median of the two data sets.

For the neuron model stimulated by piecewise constant currents we performed the Mann–Whitney U-test for univariate samples. In this case, the statistic test is corrected for continuity and is assumed to follow a normal distribution.

For all data set the null hypothesis $H_ 0$ was rejected only if $p<\alpha$, where the significance level $\alpha$ was set to 0.05.

As reported in Suppl. Table 3, it was $p>0.05$ for all the hippocampal cells and for the NEURON model, except for the interneuron cAC 97509010. For this cell, after having verified that the distribution of the differences $\textrm{data}_{\textrm{exp}}-\mathrm{data_{\textrm{mod}}}$ constitutes a sample from a normal population, we performed a Hotelling $t^2$-test to verify whether the means of $\mathrm{data_{\textrm{E}}}$ and $\mathrm{data_{\textrm{M}}}$ were equal at the significance level of 5%. For the interneuron cAC 97509010 it was $p>0.05$.

### Supplementary Information

Below is the link to the electronic supplementary material.

Suppl. Fig. 1**Plot of the derivative of** ***V*** **respect to** $I_{\text{dep}}^0$. Plot of the derivative (32) as function of $t-t^0$ obtained imposing the conditions (21) with a step of 0.01 for the parameter $\delta$ (PDF 106 KB)

Suppl. Table 1 List of parameters (PDF 101 KB)

Suppl. Table 2 Piecewise constant currents used in Fig. 8 (PDF 41 KB)

Suppl. Table 3 Model’s parameters (PDF 42 KB)

Suppl. Table 4 Initial conditions and parameters of the Monod function (PDF 38 KB)

Suppl. Table 5**Parameters of the Monod block procedure**. In the table are reported the coefficients $A_{I,II}^{j}$ and $B_{I,II}^{j}\,(j=\text{inf},\text{sup})$ of Eq. (42) that define the Monod block procedure and the corresponding values of $I_{\text{block}}^{\text{inf}}$ and $I_{\text{block}}^{\text{sup}}$ (PDF 26 KB)

Suppl. Table 6**Table of p-values of the Mann–Whitney U-test for each of the considered neurons**. *For the interneuron cAC 97509010 the null hypothesis of the Mann-Whitney U-test is rejected, then we perform a Hotelling $t^2$-test (PDF 27 KB)

Suppl. Sec. 1**Analysis for** $a>0$. Analysis of the conditions which ensure the validity of Eqs. (35) and (37) outside the range $\left[I_{\text{stim}}^{\text{min}}, I_{\text{stim}}^{\text{max}} \right]$ in the case $a>0$ (PDF 143 KB)

Suppl. Sec. 2**Analysis for** $a<0$. Analysis of the Monod function $I_{\text{adap}}^0 (\chi, I_{\text{stim}})$ for $I_{\text{stim}}$ outside the experimental range $\left[ I_{\text{stim}}^{\text{min}},I_{\text{stim}}^{\text{max}}\right]$ in the case $a<0$ (PDF 155 KB)

## Data Availability

All model and simulation files, together with a NEST implementation of the A-GLIF model using the analytical solutions, will be available in the ModelDB section of the Senselab database (http://modeldb.yale.edu/267598), and in the live papers section of EBRAINS (https://live-papers.brainsimulation.eu/).

## Footnotes

1 If $I_{\textrm{stim}}\le 0$, then the condition (26) is automatically satisfied since ${\tilde{V}}_{\textrm{th}}>-1$.

2 This can happen as a result of a strong adaptation (Migliore et al 2018) or for a depolarization block (Bianchi et al 2012).

3 In order to increase the fitting accuracy, we have relaxed the constraint $c\ge 0$ for $a>0$, since the second spike (i.e. the first one for which the $I_{\textrm{adap}}^0$ values are defined) already occurs in a range where the Monod function $I_{\textrm{adap}}^0(t_{\textrm{spk}}^+, I_\textrm{stim})$ is positive.
