# Supplementary material for: An Adaptive Generalized Leaky Integrate-and-Fire Model for Hippocampal CA1 Pyramidal Neurons and Interneurons
Source: Bull Math Biol. 2023 Oct 4;85(11):109. doi: 10.1007/s11538-023-01206-8 (PMC10550887; doi:10.1007/s11538-023-01206-8)
Supplement: Supplementary file 1 — Plot of the derivative of V respect to $I_{\text{dep}}^0$Idep0. Plot of the derivative (32) as function of $t-t^0$t-t0 obtained imposing the conditions (21) with a step of 0.01 for the parameter $\delta$δ (PDF 106 KB) [file 11538_2023_1206_MOESM1_ESM.pdf]

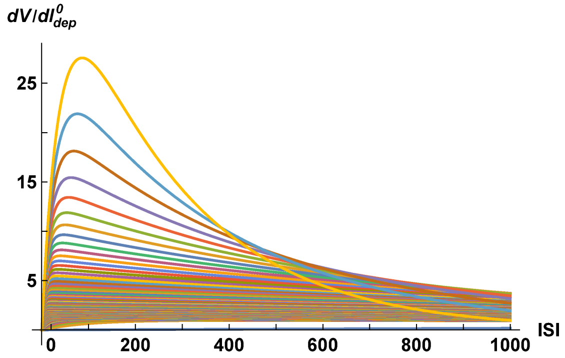

Suppl. Fig. 1 Plot of the derivative of V respect to $I^0_{\rm dep}$. Plot of the derivative (32) as function of $t - t^0$ obtained imposing the conditions (21) with a step of 0.01 for the parameter δ.
