# Supplementary material for: An Adaptive Generalized Leaky Integrate-and-Fire Model for Hippocampal CA1 Pyramidal Neurons and Interneurons
Source: Bull Math Biol. 2023 Oct 4;85(11):109. doi: 10.1007/s11538-023-01206-8 (PMC10550887; doi:10.1007/s11538-023-01206-8)
Supplement: Supplementary file 2 — List of parameters (PDF 101 KB) [file 11538_2023_1206_MOESM2_ESM.pdf]

Suppl. Table 1: List of parameters appearing in the manuscript, together with their description.

**Parameter**	**Description**
$E_{\rm L}$	resting potential
$V_{\rm r}$	reset potential
$V_{\rm th}$	threshold potential
$I_{\rm stim}$	external stimulation current
$C_{\rm m}$	membrane capacitance
$\tau_{\rm m}$	membrane time constant
$I_{\rm th}$	threshold stimulation current
$k_{\rm adap}$	adaptation constant
k_2	$I_{\rm adap}$ decay rate
k_1	$I_{\rm dep}$ decay rate
$\Delta t_{\rm ref}$	refractory interval
$t_{\rm spk}$	spike time
$t_{\rm spk}^{+}$	time instant following $t_{\rm spk} + \Delta t_{\rm ref}$
$t_{\rm start}$	last instant in which $I_{\rm stim} = 0$ or $I_{\rm stim} \leq I_{\rm th}$, while for piecewise constant stimulation currents it is updated according to Eq. (56)
T	final stimulation time
$I_{\rm dep}^{\rm start}$	initial datum of $I_{\rm dep}$ at $t_{\rm start}$
$I_{\rm adap}^{0}$	initial datum for $I_{\rm adap}$ after the first spike
$I_{\rm dep}^{0}$	initial datum for $I_{\rm dep}$ after the first spike
V^0	initial datum for the membrane potential
$\alpha = -\dfrac{I_{\rm stim}}{C_{\rm m}\, E_{\rm L}\, k_2}$	parameter of the nondimensional model
$\beta = \dfrac{k_{\rm adap}}{C_{\rm m}\, k_2^2}$	parameter of the nondimensional model
$\gamma = \dfrac{k_1}{k_2}$	parameter of the nondimensional model
$\delta = \dfrac{1}{k_2\, \tau_{\rm m}}$	parameter of the nondimensional model
$\tau = \dfrac{1}{k_2}$	parameter of the nondimensional model
K	scaling factor used in the nondimensional model
$I_{\rm stim}^{\rm min}$	minimum experimental constant stimulation current
$I_{\rm stim}^{\rm max}$	maximum experimental constant stimulation current
$F(I_{\rm stim}, \chi)$	Monod function
a, b, c, d	parameters of the Monod function
$\chi = t_{\rm spk}^{+} - t_{\rm start}$	time parameter used in the Monod function
$P(I_{\rm stim})$	plateau of the Monod function
$H(I_{\rm stim})$	superior limit for $I_{\rm adap}^{0}(\chi, I_{\rm stim})$ defined in Eq. (36)
$A_{I,II}^{\rm sup}$ (resp. $A_{I,II}^{\rm inf}$)	coefficient of $I_{\rm stim}$ related to the superior (resp. inferior) Monod block line
$B_{I,II}^{\rm sup}$ (resp. $B_{I,II}^{\rm inf}$)	known term related to the superior (resp. inferior) Monod block line
$[I_{\rm block}^{I}, I_{\rm block}^{II}]$	range of currents in which a firing block occurs
$I_{\rm fire}$	closest stimulation current above $I_{\rm block}^{II}$ or below $I_{\rm block}^{I}$ for which the firing block does not occur
$I_{\rm block}^{\rm inf} = \dfrac{I_{\rm block}^{II} + I_{\rm fire}}{2}$	when $I_{\rm fire} > I_{\rm block}^{II}$
$I_{\rm block}^{\rm sup} = \dfrac{I_{\rm block}^{I} + I_{\rm fire}}{2}$	when $I_{\rm fire} < I_{\rm block}^{I}$
$t_{\rm spk}^{\rm last}$	time of the last spike event for the current $I_{\rm stim}$
$ISI_{\rm last}$	ISI of the last spike event for the current $I_{\rm stim}$
$ISI_{\rm last}^{I}$	defined as $ISI_{\rm last}(I_{\rm block}^{I})$
$ISI_{\rm last}^{II}$	defined as $ISI_{\rm last}(I_{\rm block}^{II})$
$t_{\rm spk}^{\rm first}$	first spike time
$t_{\rm spk}^{\rm last}$	last spike time
$t_{\rm spk}^{\rm last-1}$	second-last spike time
