# Supplementary material for: An Adaptive Generalized Leaky Integrate-and-Fire Model for Hippocampal CA1 Pyramidal Neurons and Interneurons
Source: Bull Math Biol. 2023 Oct 4;85(11):109. doi: 10.1007/s11538-023-01206-8 (PMC10550887; doi:10.1007/s11538-023-01206-8)
Supplement: Supplementary file 3 — Piecewise constant currents used in Fig. 8 (PDF 41 KB) [file 11538_2023_1206_MOESM3_ESM.pdf]

<table>
<tr><th>time (ms)</th><th>0-200</th><th>200-240</th><th>240-300</th><th>300-340</th><th>340-400</th><th>400-500</th><th>500-600</th><th>600-800</th><th>800-1000</th></tr>
<tr><td>panel A</td><td>0</td><td colspan="2">600</td><td colspan="3">400</td><td>1000</td><td>0</td><td>400</td></tr>
<tr><td>panel B</td><td>0</td><td colspan="4">400</td><td colspan="2">700</td><td>200</td><td>1000</td></tr>
<tr><td>panel C</td><td>0</td><td colspan="4">600</td><td colspan="2">500</td><td>250</td><td>1000</td></tr>
<tr><td>panel D</td><td>0</td><td colspan="4">600</td><td colspan="2">500</td><td>800</td><td>1000</td></tr>
<tr><td>panel E</td><td>0</td><td>600</td><td>500</td><td>600</td><td>400</td><td colspan="3">800</td><td>1000</td></tr>
<tr><td>panel F</td><td>0</td><td colspan="4">1000</td><td colspan="2">800</td><td>400</td><td>1000</td></tr>
</table>

Suppl. Table 2: Piecewise constant currents used in Fig. 8.
