# Supplementary material for: An Adaptive Generalized Leaky Integrate-and-Fire Model for Hippocampal CA1 Pyramidal Neurons and Interneurons
Source: Bull Math Biol. 2023 Oct 4;85(11):109. doi: 10.1007/s11538-023-01206-8 (PMC10550887; doi:10.1007/s11538-023-01206-8)
Supplement: Supplementary file 4 — Model’s parameters (PDF 42 KB) [file 11538_2023_1206_MOESM4_ESM.pdf]

PYRAMIDAL NEURONS

#	NEURON ID.	E_L	V_r	V_{th}	C_m	I_{th}	τ_m	K	β	δ	τ	k_1	k_2	k_{adap}
1	95 810 005	-63.11	-46.	-34.99	2998.62	730.61	19304.94	6786.91	0.24	0.0014	27.89	0.0087	0.0359	0.9381
2	95 810 006	-62.9	-44.8	-33.71	2839.92	596.15	24332.30	7289.09	0.18	0.0010	24.51	0.0073	0.0408	0.8421
3	95 810 007	-63.	-42.62	-31.65	2640.76	595.50	12638.08	5016.46	0.24	0.0026	33.16	0.0073	0.0302	0.5794
4	95 810 008	-62.92	-41.55	-30.15	2935.78	562.35	1840.754	16582.7	0.073	0.0061	11.14	0.0066	0.0898	1.7280
5	95 810 010	-77.39	-63.48	-52.92	2998.63	585.91	96679.97	7606.69	0.24	0.00032	30.50	0.0080	0.0328	0.7868
6	95 810 011	-69.72	-53.61	-44.03	2983.24	567.37	82200.71	6190.23	0.25	0.00041	33.61	0.0074	0.0298	0.6581
7	95 810 012	-69.81	-60.71	-52.04	2993.93	15.468	85631.08	264.110	0.24	0.0092	791.4	0.0003	0.0013	0.0011
8	95 810 013	-69.89	-64.26	-51.83	2473.69	6.6709	72791.85	268.069	0.11	0.0089	644.8	0.0002	0.0016	0.0006
9	95 810 014	-69.96	-57.75	-51.3	2946.53	7.5975	7288.562	538.997	0.11	0.052	382.3	0.0003	0.0026	0.0022
10	95 810 015	-69.86	-58.17	-51.33	1889.16	10.174	18236.08	205.589	0.22	0.035	642.1	0.0003	0.0016	0.0010
11	95 810 022	-68.93	-56.47	-44.41	2978.17	201.07	81244.02	2544.03	0.22	0.00099	80.7	0.0028	0.0124	0.1021
12	95 810 023	-68.9	-56.01	-43.68	2810.88	203.32	73475.86	2390.46	0.23	0.0011	80.99	0.0029	0.0123	0.1001
13	95 810 024	-68.94	-56.57	-44.49	2638.95	205.64	78257.87	2393.69	0.24	0.00097	76.00	0.0032	0.0132	0.1112
14	95 810 025	-68.89	-55.82	-43.66	2973.05	393.53	82342.54	4506.75	0.24	0.00055	45.44	0.0053	0.0220	0.3442
15	95 810 026	-69.81	-63.3	-44.18	2098.20	403.39	30225.19	4534.27	0.24	0.0011	32.31	0.0075	0.0310	0.4894
16	95 810 027	-69.85	-62.85	-44.43	2984.37	405.49	75711.25	4711.56	0.24	0.00058	44.26	0.0054	0.0226	0.3613
17	95 810 028	-69.86	-54.29	-43.79	2654.68	552.25	64685.40	6375.63	0.23	0.00045	29.09	0.0080	0.0344	0.7295
18	95 810 029	-69.89	-52.19	-43.77	2668.84	592.85	1824.234	6725.97	0.25	0.015	27.73	0.0091	0.0361	0.8717
19	95 810 030	-65.37	-56.48	-41.83	2986.94	219.87	50233.55	2492.04	0.25	0.0016	78.36	0.0031	0.0128	0.1199
20	95 810 031	-72.95	-58.13	-56.71	2788.15	3.6663	62701.20	201.732	0.099	0.016	1008.	0.0001	0.0010	0.0003
21	95 810 032	-59.82	-62.62	-42.37	1023.88	5.0945	11678.68	84.1546	0.27	0.062	727.7	0.0004	0.0014	0.0005
22	95 810 033	-65.85	-58.91	-45.36	2753.39	7.2598	35118.23	295.181	0.098	0.017	614.5	0.0002	0.0016	0.0007
23	95 810 037	-65.88	-66.66	-51.03	1996.50	7.1148	98786.96	149.401	0.22	0.0089	880.3	0.0003	0.0011	0.0006
24	95 810 038	-65.95	-66.74	-51.1	2907.63	4.8747	26782.56	483.352	0.062	0.015	396.7	0.0002	0.0025	0.0011
25	95 810 039	-65.8	-66.58	-51.02	2901.24	8.5130	59415.43	194.401	0.21	0.017	982.2	0.0002	0.0010	0.0006
26	95 810 040	-65.99	-66.93	-51.46	2795.08	3.4871	21414.76	262.075	0.095	0.033	703.9	0.0001	0.0014	0.0005
27	95 810 041	-65.97	-55.68	-46.08	2993.05	204.34	75004.83	2837.39	0.24	0.00093	69.56	0.0035	0.0144	0.1486
28	95 817 003	-69.94	-58.41	-49.46	2961.59	582.87	22931.88	8090.58	0.25	0.0011	25.60	0.0097	0.0391	1.1180
29	95 817 004	-59.85	-45.75	-42.82	2993.98	200.35	89032.42	2909.89	0.24	0.00069	61.57	0.0039	0.0162	0.1916
30	95 817 005	-74.04	-54.59	-53.08	2970.50	553.88	30136.08	8116.00	0.24	0.00090	27.11	0.0089	0.0369	0.9788
31	95 817 006	-74.03	-54.07	-52.	2992.12	569.23	73773.30	9222.80	0.21	0.00033	24.03	0.0087	0.0416	1.0790
32	95 817 007	-69.63	-50.18	-44.5	2946.98	362.39	58758.02	4063.71	0.25	0.00086	50.49	0.0049	0.0198	0.2867
33	95 817 008	-68.9	-50.46	-43.78	2894.86	349.32	42334.75	4095.85	0.24	0.0011	48.68	0.0048	0.0205	0.2874
34	95 822 000	-69.87	-65.26	-53.27	2025.15	1.9530	52167.20	197.393	0.057	0.014	716.9	0.0001	0.0014	0.0002
35	95 822 001	-69.95	-65.72	-53.83	2911.84	1.4517	39090.49	126.451	0.093	0.041	1610.	0.0001	0.0006	0.0001
36	95 822 002	-69.33	-52.41	-54.32	2886.04	0.47183	32764.57	57.6564	0.15	0.11	3469.	0.0000	0.0003	0.0000
37	95 822 003	-69.77	-51.75	-54.6	2848.62	7.4124	81108.61	172.290	0.21	0.014	1153.	0.0002	0.0009	0.0005
38	95 822 005	-70.07	-61.25	-55.2	2877.83	26.133	10955.36	839.157	0.17	0.022	240.3	0.0007	0.0042	0.0084
39	95 822 006	-69.17	-49.96	-52.77	2971.35	6.3183	86007.72	167.815	0.17	0.014	1225.	0.0001	0.0008	0.0003
40	95 822 009	-70.06	-74.29	-58.84	2965.22	31.401	54894.25	1286.07	0.16	0.0029	161.5	0.0010	0.0062	0.0178
41	95 822 010	-67.78	-65.34	-50.96	2976.80	514.87	57340.19	8502.88	0.25	0.00041	23.72	0.0103	0.0421	1.2960
42	95 822 011	-67.93	-63.9	-49.82	2898.94	708.92	50054.51	11801.2	0.23	0.00033	16.69	0.0135	0.0599	2.3520
43	95 824 000	-70.62	-69.45	-63.72	2869.10	2.0686	24031.21	268.608	0.11	0.031	754.4	0.0001	0.0013	0.0006
44	95 824 004	-77.98	-55.7	-57.47	2960.72	375.22	90396.49	5835.43	0.24	0.00044	39.57	0.0062	0.0253	0.4630
45	95 824 006	-70.88	-64.66	-58.76	2899.23	4.4304	29535.53	677.396	0.051	0.010	303.3	0.0002	0.0033	0.0016
46	95 831 000	-76.51	-56.3	-50.9	2989.67	335.69	30168.61	4069.29	0.25	0.0019	56.20	0.0044	0.0178	0.2352
47	95 831 001	-70.74	-59.78	-56.38	2792.16	15.659	51421.79	608.047	0.13	0.0063	324.9	0.0004	0.0031	0.0036
48	95 831 002	-68.33	-54.08	-42.65	2981.65	376.15	89098.87	4528.40	0.22	0.00050	44.98	0.0049	0.0222	0.3270
49	95 831 003	-72.	-53.24	-43.81	2946.89	571.66	89773.23	8060.26	0.18	0.00029	26.32	0.0069	0.0380	0.7744
50	95 831 004	-65.98	-56.64	-45.	2669.05	6.5074	72624.76	212.799	0.11	0.011	827.8	0.0001	0.0012	0.0004
51	95 912 004	-66.17	-63.63	-49.66	2823.86	15.480	83967.84	383.136	0.17	0.0058	487.8	0.0003	0.0021	0.0020
52	95 912 005	-77.4	-57.55	-59.	981.518	3.5945	89792.55	253.717	0.065	0.0033	299.3	0.0002	0.0033	0.0007
53	95 912 006	-76.56	-60.95	-60.06	2326.93	6.0786	26535.76	201.116	0.17	0.033	885.8	0.0002	0.0011	0.0005
54	95 912 007	-68.7	-64.48	-54.11	1302.95	1.6780	52407.01	71.6487	0.14	0.024	1249.	0.0001	0.0008	0.0001
55	95 914 001	-66.42	-65.22	-50.97	2912.53	367.42	49312.68	6627.68	0.24	0.00059	29.20	0.0082	0.0343	0.8160
56	95 914 002	-73.74	-52.24	-46.47	2432.59	206.78	44839.68	2303.27	0.24	0.0017	77.89	0.0031	0.0128	0.0980
57	95 914 003	-73.91	-58.16	-46.15	2941.26	363.47	8227.682	4042.41	0.25	0.0065	53.80	0.0046	0.0186	0.2499
58	95 914 004	-74.09	-51.75	-45.42	2937.38	376.77	95878.42	4426.13	0.22	0.00051	49.19	0.0045	0.0203	0.2681

INTERNEURONS -- BAC

1	96 711 008	-70.54	-37.3	-47.37	2875.63	203.21	40946.81	2298.26	0.19	0.0022	88.29	0.0022	0.0113	0.0703
2	97 911 000	-82.51	-49.29	-55.03	2963.79	538.94	50524.51	5376.89	0.25	0.00090	45.47	0.0055	0.0220	0.3578
3	97 911 001	-83.78	-49.29	-55.41	2694.16	429.32	79162.48	4369.53	0.24	0.00065	51.64	0.0046	0.0194	0.2418
4	97 911 002	-75.72	-42.27	-49.73	2949.44	213.08	77672.58	2071.73	0.23	0.0014	107.8	0.0022	0.0093	0.0595
5	99 111 000	-73.98	-50.71	-60.19	2957.59	204.08	43170.78	2829.02	0.23	0.0018	77.36	0.0030	0.0129	0.1144
6	99 111 001	-74.36	-49.25	-58.22	2995.82	206.31	66549.29	2880.45	0.21	0.0012	77.35	0.0028	0.0129	0.1069
7	99 111 002	-75.16	-50.43	-59.6	2790.36	20.759	20685.47	468.783	0.16	0.022	447.5	0.0004	0.0022	0.0022

INTERNEURONS -- CAC

1	97 428 000	-74.01	-34.98	-49.48	2939.66	191.54	2169.407	2135.48	0.22	0.047	101.9	0.0021	0.0098	0.0616
2	97 428 001	-75.19	-29.3	-40.33	792.686	0.69101	931.1111	83.5431	0.78	0.77	713.4	0.0011	0.0014	0.0012
3	97 509 008	-75.59	-40.39	-57.8	2311.95	375.80	93875.21	3315.32	0.24	0.00056	52.69	0.0046	0.0190	0.2032
4	97 509 009	-69.11	-45.04	-59.6	2980.73	598.49	72226.15	7143.61	0.24	0.00040	28.84	0.0084	0.0347	0.8631
5	97 509 010	-68.97	-40.9	-56.5	2679.44	394.60	28449.60	7348.71	0.13	0.00088	25.16	0.0053	0.0398	0.5674
6	97 509 011	-69.5	-45.92	-60.83	2871.99	415.67	15755.71	5069.04	0.24	0.0025	39.38	0.0062	0.0254	0.4529
7	98 205 021	-78.77	-39.75	-59.82	2832.86	281.89	66859.41	2414.00	0.24	0.0014	92.41	0.0026	0.0108	0.0787
8	98 205 022	-80.81	-43.08	-64.75	2913.93	234.38	37534.88	2223.32	0.23	0.0028	105.9	0.0022	0.0094	0.0595
9	98 205 024	-76.29	-39.11	-61.76	2972.13	203.95	39781.13	1883.95	0.23	0.0030	120.4	0.0019	0.0083	0.0463
10	98 205 025	-78.18	-39.62	-60.68	2992.97	213.32	38167.21	1777.47	0.25	0.0034	131.7	0.0019	0.0076	0.0426

INTERNEURONS -- CNAC

1	95 817 000	-74.36	-53.61	-71.01	2993.71	396.96	13910.13	6140.47	0.23	0.0026	36.26	0.0065	0.0276	0.5345
2	95 817 001	-74.58	-55.36	-72.08	2979.62	398.32	16620.99	6642.73	0.24	0.0020	33.45	0.0070	0.0299	0.6262
3	95 817 002	-74.63	-56.21	-73.68	2826.67	401.79	10625.26	6636.21	0.25	0.0030	31.79	0.0078	0.0315	0.6952
4	97 717 005	-68.51	-36.4	-52.19	2984.47	578.10	71976.11	5099.57	0.24	0.00056	40.09	0.0060	0.0249	0.4502
5	98 513 011	-73.79	-44.68	-62.02	2969.00	227.12	70719.82	2684.82	0.22	0.0012	81.63	0.0026	0.0123	0.0963
6	99 111 004	-80.79	-40.38	-55.09	2683.82	45.924	47390.64	476.693	0.20	0.0096	455.0	0.0004	0.0022	0.0026
7	99 111 006	-84.61	-40.26	-54.2	2722.84	69.87	3124.192	756.480	0.27	0.097	304.6	0.0009	0.0033	0.0080
8	98D15008	-73.03	-34.44	-49.98	2803.35	580.18	92651.58	5156.28	0.21	0.00043	39.69	0.0054	0.0252	0.3802
9	98D15009	-73.52	-35.4	-48.45	2966.74	622.26	52222.11	4932.33	0.24	0.00085	44.21	0.0055	0.0226	0.3706

NEURON

1	NEURON	-72.5	-52	-65	2047.42	301.00	3310.462	4526.33	0.25	0.0099	32.79	0.0075	0.0305	0.4668

LAYER 5 VISUAL CORTEX NEURON

1	476048909	-66.2	-50.35	-36.97	2342.76	152.15	8383.019	1481.05	0.25	0.012	104.7	0.0023	0.0095	0.0524
