# Supplementary material for: An Adaptive Generalized Leaky Integrate-and-Fire Model for Hippocampal CA1 Pyramidal Neurons and Interneurons
Source: Bull Math Biol. 2023 Oct 4;85(11):109. doi: 10.1007/s11538-023-01206-8 (PMC10550887; doi:10.1007/s11538-023-01206-8)
Supplement: Supplementary file 5 — Initial conditions and parameters of the Monod function (PDF 38 KB) [file 11538_2023_1206_MOESM5_ESM.pdf]

Out[•]=

PYRAMIDAL NEURONS

#	NEURON ID.	I_{dep}^{start}	I_{dep}^{0}	a	b	c	d
1	95 810 005	0.012	0.64	6.91×10^{13}	−0.00036	0.0847	4.49×10^{15}
2	95 810 006	0.020	0.44	6.39	−0.000494	−3.15	1.08
3	95 810 007	0.019	0.14	2.4×10^{12}	0.00113	0.0574	1.21×10^{15}
4	95 810 008	0.0044	0.37	4.81×10^{13}	−0.000585	−0.708	1.89×10^{15}
5	95 810 010	0.0070	0.37	44.7	−0.0000338	−42.9	0.081
6	95 810 011	0.013	0.57	8.79	−0.000814	−0.198	273.
7	95 810 012	0.44	53.	75.5	−0.000239	−10.6	15.2
8	95 810 013	0.73	92.	138.	−0.000176	−22.8	14.7
9	95 810 014	0.48	58.	87.7	−0.000262	−11.4	15.1
10	95 810 015	0.36	92.	176.	−0.0000395	−74.9	2.42
11	95 810 022	0.0087	1.4	7.47	−0.000322	−1.19	93.4
12	95 810 023	0.0074	1.1	6.02	−0.000154	−1.28	81.5
13	95 810 024	0.0082	1.3	6.51	−0.00019	−1.44	64.3
14	95 810 025	0.037	0.56	3.53	0.000154	0.177	507.
15	95 810 026	0.0086	0.75	3.91	−0.000504	−0.327	78.2
16	95 810 027	0.012	1.2	8.59	−0.00117	−0.378	105.
17	95 810 028	0.025	0.69	4.00	−0.00077	−0.184	92.2
18	95 810 029	0.15	0.55	4.94	−0.00104	−0.0797	174.
19	95 810 030	0.024	3.0	8.32	−0.000429	−0.662	91.4
20	95 810 031	0.21	82.	50700.	3.88×10^{-7}	−50600.	0.0255
21	95 810 032	0.54	82.	8300.	2.44×10^{-6}	−8210.	0.0378
22	95 810 033	0.77	90.	163.	−0.000131	−35.9	12.2
23	95 810 037	0.31	95.	163.	−0.0000401	−44.6	9.34
24	95 810 038	0.59	81.	142.	−0.000152	−18.0	31.4
25	95 810 039	0.43	59.	100.	−0.00012	−14.5	35.4
26	95 810 040	0.64	90.	161.	−0.00012	−25.1	38.4
27	95 810 041	0.0077	1.7	6.09	−0.0000739	−1.76	41.4
28	95 817 003	0.0017	0.21	244000.	3.06×10^{-9}	−244000.	0.000103
29	95 817 004	0.0064	0.11	5.16	0.000185	−4.42	5.27
30	95 817 005	0.0098	0.52	1.88×10^{8}	0.000170	0.0348	2.41×10^{10}
31	95 817 006	0.013	0.71	63.	-9.77×10^{-6}	−61.7	0.00639
32	95 817 007	0.0081	1.2	3.79	−0.0000382	−0.443	113.
33	95 817 008	0.0041	0.59	6.42	−0.000162	−0.46	541.
34	95 822 000	0.24	98.	23700.	1.77×10^{-6}	−23600.	0.0803
35	95 822 001	0.54	79.	177.	0.0000507	−29.8	43.9
36	95 822 002	0.85	6.6	2.07×10^{7}	5.94×10^{-9}	-2.07×10^{7}	0.000031
37	95 822 003	0.20	15.	5.09×10^{6}	6.18×10^{-9}	-5.09×10^{6}	0.0000188
38	95 822 005	0.10	26.	56.9	0.0000333	−28.5	3.53
39	95 822 006	0.57	61.	126.	−0.000163	−14.5	45.3
40	95 822 009	0.066	27.	83.9	−0.0000856	−49.2	0.934
41	95 822 010	0.0057	0.081	5.99×10^{14}	−0.00115	−0.0839	2.04×10^{16}
42	95 822 011	0.011	0.11	1.84	−0.000693	−0.0471	77.9
43	95 824 000	0.15	39.	74.8	0.000153	−20.1	26.8
44	95 824 004	0.013	0.14	3.58×10^{12}	0.00175	0.103	3.64×10^{15}
45	95 824 006	0.51	94.	135.	0.0000378	−37.9	5.74
46	95 831 000	0.010	1.5	4.23	−0.000369	−0.353	110.
47	95 831 001	0.22	49.	1.99×10^{7}	2.89×10^{-10}	-1.99×10^{7}	5.87×10^{-6}
48	95 831 002	0.016	0.86	3.88	−0.000648	−0.134	203.
49	95 831 003	0.0054	0.35	4.67	−0.000982	−0.0384	365.
50	95 831 004	0.32	99.	337.	−0.0000154	−201.	6.73
51	95 912 004	0.090	54.	173.	−0.0000398	−104.	6.83
52	95 912 005	0.11	9.0	47.	0.000584	−29.	18.7
53	95 912 006	0.14	86.	528.	-2.93×10^{-6}	−434.	1.68
54	95 912 007	0.63	58.	116.	0.000302	−32.8	22.7
55	95 914 001	0.0069	0.36	2.29	0.000236	−1.69	5.08
56	95 914 002	0.016	0.60	4.55	−0.000361	−0.469	83.4
57	95 914 003	0.017	1.0	3.47	−0.00023	−0.291	64.6
58	95 914 004	0.026	0.99	4.65	−0.00023	−0.027	164.

INTERNEURONS -- BAC

1	96 711 008	0.96	11.	2060.	-4.04×10^{-6}	−2050.	0.0103
2	97 911 000	0.048	13.	6.89	0.001	−1.21	9.65
3	97 911 001	0.030	15.	4.50×10^{6}	-6.35×10^{-10}	-4.50×10^{6}	0.0000204
4	97 911 002	0.070	44.	5.05×10^{6}	8.44×10^{-10}	-5.05×10^{6}	0.0000433
5	99 111 000	0.078	21.	43.3	−0.000817	7.68	80.0
6	99 111 001	0.046	15.	24.5	−0.000376	2.44	87.5
7	99 111 002	0.98	79.	130.	−0.000612	15.3	71.4

INTERNEURONS -- CAC

1	97 428 000	0.31	92.	40.4	−0.00137	71.1	10
2	97 428 001	0.92	78.	32.4	−0.00128	68.3	60.0
3	97 509 008	0.018	6.3	183.	−0.0000709	−169.	0.188
4	97 509 009	0.24	0.28	0.956	0.000411	0.149	264.
5	97 509 010	0.037	14.	37.6	−0.000890	−0.109	32.4
6	97 509 011	0.024	1.6	64.8	−0.00333	−0.658	25.0
7	98 205 021	0.21	25.	39.4	−0.00130	3.81	22.9
8	98 205 022	0.14	20.	46.9	−0.00138	−0.510	73.1
9	98 205 024	0.094	21.	147.	−0.00259	−1.97	107.
10	98 205 025	0.096	23.	93.7	−0.00191	−2.94	49.9

INTERNEURONS -- CNAC

1	95 817 000	0.10	7.0	34.3	−0.00198	−3.5	3.56
2	95 817 001	0.16	5.8	29.2	−0.00249	−0.204	29.8
3	95 817 002	0.11	5.5	44.4	−0.00308	0.210	49.2
4	97 717 005	0.053	7.0	123.	−0.000173	−103.	0.0277
5	98 513 011	0.030	13.	60.6	−0.000563	−33.5	0.736
6	99 111 004	0.70	98.	373.	−0.00817	−0.142	10
7	99 111 006	0.40	88.	164.	−0.00151	−29.1	2.87
8	98D15008	0.0099	7.5	92.3	−0.000134	−75.6	0.698
9	98D15009	0.76	3.4	−526.	−0.515	1.75	3.95

NEURON

1	NEURON	0.018	1.0	14.3	−0.00211	0.0609	225.5

LAYER 5 VISUAL CORTEX NEURON

1	476048909	0.029	1.1	21.7	−0.00672	−0.01669	412.7
