# Supplementary material for: An Adaptive Generalized Leaky Integrate-and-Fire Model for Hippocampal CA1 Pyramidal Neurons and Interneurons
Source: Bull Math Biol. 2023 Oct 4;85(11):109. doi: 10.1007/s11538-023-01206-8 (PMC10550887; doi:10.1007/s11538-023-01206-8)
Supplement: Supplementary file 6 — Parameters of the Monod block procedure. In the table are reported the coefficients $A_{I,II}^{j}$AI,IIj and $B_{I,II}^{j}\,(j=\text{inf},\text{sup})$BI,IIj(j=inf,sup) of Eq. (42) that define the Monod block procedure and the corresponding values of $I_{\text{block}}^{\text{inf}}$Iblockinf and $I_{\text{block}}^{\text{sup}}$Iblocksup (PDF 26 KB) [file 11538_2023_1206_MOESM6_ESM.pdf]

PYRAMIDAL NEURONS							
#	NEURON ID.	I^{sup}_{block}	$A^{sup}_{I,II}$	$B^{sup}_{I,II}$	I^{inf}_{block}	$A^{inf}_{I,II}$	$B^{inf}_{I,II}$
1	95 810 005	--	--	--	900.0	1.141	-711.6
2	95 810 006	--	--	--	--	--	--
3	95 810 007	900.0	-1.4303	1658.1	--	--	--
4	95 810 008	700.0	0.1025	121.65	--	--	--
5	95 810 010	900.0	-0.68475	852.7	--	--	--
6	95 810 011	--	--	--	--	--	--
7	95 810 012	--	--	--	--	--	--
8	95 810 013	--	--	--	--	--	--
9	95 810 014	--	--	--	--	--	--
10	95 810 015	--	--	--	300.0	0.835	52.4
11	95 810 022	--	--	--	--	--	--
12	95 810 023	--	--	--	--	--	--
13	95 810 024	--	--	--	--	--	--
14	95 810 025	--	--	--	500.0	0.8655	-66.75
15	95 810 026	--	--	--	--	--	--
16	95 810 027	--	--	--	700.0	0.5935	-102.9
17	95 810 028	--	--	--	--	--	--
18	95 810 029	--	--	--	--	--	--
19	95 810 030	--	--	--	--	--	--
20	95 810 031	--	--	--	300.0	0.96325	-51.95
21	95 810 032	--	--	--	300.0	0.42125	143.85
22	95 810 033	--	--	--	300.0	1.6185	-138.95
23	95 810 037	--	--	--	--	--	--
24	95 810 038	--	--	--	--	--	--
25	95 810 039	--	--	--	--	--	--
26	95 810 040	--	--	--	--	--	--
27	95 810 041	--	--	--	--	--	--
28	95 817 003	--	--	--	--	--	--
29	95 817 004	--	--	--	--	--	--
30	95 817 005	800	0.0675	58.65	800	-0.33	376.65
31	95 817 006	800	-0.07075	207.9	800	0.09775	73.1
32	95 817 007	--	--	--	--	--	--
33	95 817 008	--	--	--	--	--	--
34	95 822 000	--	--	--	500.0	0.011	194.95
35	95 822 001	--	--	--	300.0	1.7768	-193.1
36	95 822 002	900.0	-1.436	1611.2	500.0	0.443	-46.15
37	95 822 003	377	0.01275	130.65	377	0.519	-60.
38	95 822 005	900.0	-0.07275	403.95	700.0	0.4005	7.05
39	95 822 006	--	--	--	500.0	-0.4665	321.15
40	95 822 009	600.0	-0.13125	232.5	600.0	0.0735	15.3
41	95 822 010	900.0	-1.9928	2075.	700.0	2.1225	-1217.3
42	95 822 011	900.0	-0.42025	503.7	900.0	-0.42025	503.7
43	95 824 000	--	--	--	--	--	--
44	95 824 004	700.0	-0.46625	612.45	--	--	--
45	95 824 006	--	--	--	--	--	--
46	95 831 000	--	--	--	--	--	--
47	95 831 001	492	-0.018	173.95	492	0.4635	-63.
48	95 831 002	--	--	--	--	--	--
49	95 831 003	--	--	--	--	--	--
50	95 831 004	--	--	--	300.0	0.58975	86.05
51	95 912 004	--	--	--	300.0	1.141	-130.85
52	95 912 005	--	--	--	--	--	--
53	95 912 006	--	--	--	--	--	--
54	95 912 007	--	--	--	--	--	--
55	95 914 001	--	--	--	900.0	-0.0875	160.7
56	95 914 002	700.0	0.15125	77.	--	--	--
57	95 914 003	500.0	0.1175	112.45	--	--	--
58	95 914 004	700.0	-0.024	275.45	700.0	0.0985	124.85
INTERNEURONS -- BAC							
1	96 711 008	700.0	0.74575	-436.3	--	--	--
2	97 911 000	--	--	--	900.0	0.336	-173.6
3	97 911 001	--	--	--	700.0	1.8775	-1092.5
4	97 911 002	900.0	-0.19625	553.3	700.0	1.2095	-447.65
5	99 111 000	500.0	0.26	60.15	--	--	--
6	99 111 001	--	--	--	700.0	0.38775	-10.4
7	99 111 002	700.0	0.11125	221.85	500.0	-0.8155	447.05
INTERNEURONS -- CAC							
1	97 428 000	--	--	--	300.0	0.95975	32.
2	97 428 001	--	--	--	--	--	--
3	97 509 008	900.0	-0.08575	460.7	--	--	--
4	97 509 009	--	--	--	--	--	--
5	97 509 010	--	--	--	--	--	--
6	97 509 011	900.0	-0.57425	738.	700.0	0.714	-292.6
7	98 205 021	700.0	-1.0095	996.8	--	--	--
8	98 205 022	700.0	-0.22075	542.85	--	--	--
9	98 205 024	--	--	--	500.0	0.80075	-81.2
10	98 205 025	--	--	--	500.0	0.89825	-144.65
INTERNEURONS -- CNAC							
1	95 817 000	--	--	--	700.0	0.896	-201.5
2	95 817 001	--	--	--	700.0	0.47325	31.2
3	95 817 002	--	--	--	--	--	--
4	97 717 005	--	--	--	--	--	--
5	98 513 011	--	--	--	--	--	--
6	99 111 004	300.0	-0.10325	331.8	--	--	--
7	99 111 006	500.0	-0.1675	378.35	300.0	0.68075	123.8
8	98D15008	--	--	--	700.0	0.04275	358.35
9	98D15009	--	--	--	--	--	--
NEURON							
1	NEURON	--	--	--	700.0	0.68	-190.
LAYER 5 VISUAL CORTEX NEURON							
1	476048909	--	--	--	280.	7.66075	1321.26
