# Supplementary material for: An Adaptive Generalized Leaky Integrate-and-Fire Model for Hippocampal CA1 Pyramidal Neurons and Interneurons
Source: Bull Math Biol. 2023 Oct 4;85(11):109. doi: 10.1007/s11538-023-01206-8 (PMC10550887; doi:10.1007/s11538-023-01206-8)
Supplement: Supplementary file 7 — Table of p-values of the Mann–Whitney U-test for each of the considered neurons. *For the interneuron cAC 97509010 the null hypothesis of the Mann-Whitney U-test is rejected, then we perform a Hotelling $t^2$t2-test (PDF 27 KB) [file 11538_2023_1206_MOESM7_ESM.pdf]

PYRAMIDAL NEURONS

NEURON ID	95 810 005	95 810 006	95 810 007	95 810 008	95 810 010	95 810 011	95 810 012
p-value	0.8976	0.6095	0.983	0.9541	0.5977	0.6747	0.9786
NEURON ID	95 810 013	95 810 014	95 810 015	95 810 022	95 810 023	95 810 024	95 810 025
p-value	0.9938	0.9634	0.8806	0.827	0.9489	0.6236	0.9633
NEURON ID	95 810 026	95 810 027	95 810 028	95 810 029	95 810 030	95 810 031	95 810 032
p-value	0.691	0.8964	0.7784	0.8993	0.8572	0.8981	0.5527
NEURON ID	95 810 033	95 810 037	95 810 038	95 810 039	95 810 040	95 810 041	95 817 003
p-value	0.743	0.951	0.9814	0.8039	0.918	0.8884	0.8208
NEURON ID	95 817 004	95 817 005	95 817 006	95 817 007	95 817 008	95 822 000	95 822 001
p-value	0.4466	0.9999	0.9928	0.7643	0.8815	0.9888	0.9674
NEURON ID	95 822 002	95 822 003	95 822 005	95 822 006	95 822 009	95 822 010	95 822 011
p-value	0.991	0.4436	0.6812	0.5597	0.9062	0.972	0.9776
NEURON ID	95 824 000	95 824 004	95 824 006	95 831 000	95 831 001	95 831 002	95 831 003
p-value	0.9607	0.9078	0.9997	0.9107	0.7766	0.9713	0.9438
NEURON ID	95 831 004	95 912 004	95 912 005	95 912 006	95 912 007	95 914 001	95 914 002
p-value	0.9076	0.934	0.9997	0.9912	0.9746	0.229	0.8395
NEURON ID	95 914 003	95 914 004					
p-value	0.9197	0.9521					

INTERNEURONS -- BAC

NEURON ID	96 711 008	97 911 000	97 911 001	97 911 002	99 111 000	99 111 001	99 111 002
p-value	0.4322	0.2462	0.06776	0.3647	0.6132	0.6433	0.9832

INTERNEURONS -- CAC

NEURON ID	97 428 000	97 428 001	97 509 008	97 509 009	97 509 010 *	97 509 011	98 205 021
p-value	0.05848	0.7148	0.1837	0.939	0.02443	0.8539	0.9761
NEURON ID	98 205 022	98 205 024	98 205 025				
p-value	0.991	0.0567 *	0.9484				

INTERNEURONS -- CNAC

NEURON ID	95 817 000	95 817 001	95 817 002	97 717 005	98 513 011	99 111 004	99 111 006
p-value	0.8857	0.7375	0.7322	0.3555	0.8205	0.07709	0.8737
NEURON ID	98D15008						
p-value	0.7246						

NEURON

NEURON ID	NEURON						
p-value	0.999937						

LAYER 5 VISUAL CORTEX NEURON

NEURON ID	476048909						
p-value	0.8288						
