# Supplementary material for: An Adaptive Generalized Leaky Integrate-and-Fire Model for Hippocampal CA1 Pyramidal Neurons and Interneurons
Source: Bull Math Biol. 2023 Oct 4;85(11):109. doi: 10.1007/s11538-023-01206-8 (PMC10550887; doi:10.1007/s11538-023-01206-8)
Supplement: Supplementary file 8 — Analysis for $a>0$a>0. Analysis of the conditions which ensure the validity of Eqs. (35) and (37) outside the range $\left[I_{\text{stim}}^{\text{min}}, I_{\text{stim}}^{\text{max}} \right]$Istimmin,Istimmax in the case $a>0$a>0 (PDF 143 KB) [file 11538_2023_1206_MOESM8_ESM.pdf]

Suppl. Section 1: $a > 0$

In the following, we analyse the conditions which ensure the validity of Eq. (35) and Eq. (37) outside the range $\left[I_{\text{stim}}^{\min}, I_{\text{stim}}^{\max}\right]$ in the case $a > 0$.

(1.1) $b < 0$. We note that the NEURON model considered here to test our A-GLIF model falls within this case. According to Prop. 1, under these conditions we have that $I_{\text{adap}}^{0}(\chi, I_{\text{stim}})$ is monotonically increasing w.r.t. χ and monotonically decreasing w.r.t. I_{stim}. The plateau function $P(I_{\text{stim}})$ is also monotonically decreasing. We distinguish two further subcases:

- If $c \geq 0$ and $c < I_{\text{adap}}^{0}(\chi, I_{\text{stim}}) < P(I_{\text{stim}}) < H(I_{\text{stim}})$ $\forall I_{\text{stim}} \in \left[I_{\text{stim}}^{\min}, I_{\text{stim}}^{\max}\right]$, for all the stimulation currents above the experimental range, the following inequality holds

$$c < F(\chi, I_{\text{stim}}) < P(I_{\text{stim}}) < H(I_{\text{stim}}) \quad \forall I_{\text{stim}} > I_{\text{stim}}^{\max}.$$

  In this case, both the positivity property of the Monod function in Eq. (35) and the threshold condition in Eq. (37) are then automatically satisfied. As for the stimulation currents below the experimental range, we have that

$$c < I_{\text{adap}}^{0}(\chi, I_{\text{stim}}) < P(I_{\text{stim}}) < P(I_{\text{stim}}^{\min}) \quad \forall I_{\text{stim}} < I_{\text{stim}}^{\min}.$$

  Here the positivity of the Monod function still holds; however, since the function $H(I_{\text{stim}})$ is monotonically increasing, it is not a priori guaranteed that $P(I_{\text{stim}}) < H(I_{\text{stim}})$, $\forall I_{\text{stim}} < I_{\text{stim}}^{\min}$. In our optimization procedure we accept that the threshold condition (37) is initially violated, as translations of the Monod function to satisfy Eq. (37) potentially violates the positivity constraint (35).

- If $c < 0$ and $0 \leq I_{\text{adap}}^{0}(\chi, I_{\text{stim}}) < P(I_{\text{stim}}) < H(I_{\text{stim}})$ $\forall I_{\text{stim}} \in \left[I_{\text{stim}}^{\min}, I_{\text{stim}}^{\max}\right]$ and $\forall \chi \in \left[t_{\text{spk}}^{\text{first}}\left(I_{\text{stim}}\right), t_{\text{spk}}^{\text{last}-1}\left(I_{\text{stim}}\right)\right]$, we have

$$I_{\text{adap}}^{0}(\chi, I_{\text{stim}}) < P(I_{\text{stim}}) < H(I_{\text{stim}}), \quad \forall I_{\text{stim}} > I_{\text{stim}}^{\max}.$$

  However, the plateau function $P(I_{\text{stim}})$ is positive only when $I_{\text{stim}} < \log\left(-c/a\right)/b$; more specifically

$$0 < P(I_{\text{stim}}) < H(I_{\text{stim}}), \quad \forall I_{\text{stim}} < \frac{\log\left(-c/a\right)}{b}.$$

  In this case, the positivity of the Monod function is guaranteed only for a specific range of stimulation currents. Outside the experimental range of stimulation currents, it can also occur that the Monod function is negative $\forall \chi > 0$. In this case, the function $I_{\text{adap}}^{0}(\chi, I_{\text{stim}})$ must be modified in order to satisfy the positivity condition (35) and, eventually, also the threshold condition (37). The easiest way to

achieve this is to translate the Monod function along the vertical axis as reported in Eqs.(49)–(51).

(1.2) $b > 0$. In this case, according to Prop. 1, we have that $I^0_{\text{adap}}(\chi, I_{\text{stim}})$ is monotonically increasing w.r.t. χ and I_{stim}. Moreover, the plateau function $P(I_{\text{stim}})$ is also monotonically increasing. We observe that:

– If $c \geq 0$ and $c < I^0_{\text{adap}}(\chi, I_{\text{stim}}) < P(I_{\text{stim}}) < H(I_{\text{stim}})$ $\forall I_{\text{stim}} \in \left[I^{\min}_{\text{stim}}, I^{\max}_{\text{stim}}\right]$, for all the stimulation currents above the experimental range the following inequality holds

$$c < I^0_{\text{adap}}(\chi, I_{\text{stim}}) < P(I_{\text{stim}}) \quad \forall I_{\text{stim}} > I^{\max}_{\text{stim}} \quad \forall I_{\text{stim}} < I^{\min}_{\text{stim}}.$$

In this case, Eq. (35) is hence automatically verified. However, Eq. (37) may not hold outside the experimental range of stimulation currents for all $\chi > 0$. In order to satisfy Eq. (37), it is possible to proceed with a translation of the Monod function as in Eq. (49), as long as this does not violate the positivity constraint (35).

– If $c < 0$ and $0 \leq I^0_{\text{adap}}(\chi, I_{\text{stim}}) < P(I_{\text{stim}}) < H(I_{\text{stim}})$ $\forall I_{\text{stim}} \in \left[I^{\min}_{\text{stim}}, I^{\max}_{\text{stim}}\right]$ and $\forall \chi \in \left[t^{\text{first}}_{\text{spk}}\left(I_{\text{stim}}\right), t^{\text{last}-1}_{\text{spk}}\left(I_{\text{stim}}\right)\right]$, we have that, for all $I_{\text{stim}} > I^{\max}_{\text{stim}}$ and for all $\chi \in \left[t^{\text{first}}_{\text{spk}}\left(I_{\text{stim}}\right), t^{\text{last}-1}_{\text{spk}}\left(I_{\text{stim}}\right)\right]$,

$$I^0_{\text{adap}}(\chi, I_{\text{stim}}) < P(I_{\text{stim}}).$$

In this case, neither Eq. (35) nor Eq. (37) are verified a priori. It is possible to apply the translation defined in Eq. (51) to ensure the validity of Eq. (35); however, it might happen that Eq. (37) is not verified for all times.

As for $I_{\text{stim}} < I^{\min}_{\text{stim}}$, the Monod function might become negative. In this case, one possibility to restore the positivity of the Monod function is to modify it via a translation as the one defined in Eq. (49)-(51). As we do not encounter situations with a negative Monod function for the neurons studied in this paper, we cannot test the validity of this approach.
