# Supplementary material for: An Adaptive Generalized Leaky Integrate-and-Fire Model for Hippocampal CA1 Pyramidal Neurons and Interneurons
Source: Bull Math Biol. 2023 Oct 4;85(11):109. doi: 10.1007/s11538-023-01206-8 (PMC10550887; doi:10.1007/s11538-023-01206-8)
Supplement: Supplementary file 9 — Analysis for $a<0$a<0. Analysis of the Monod function $I_{\text{adap}}^0 (\chi, I_{\text{stim}})$Iadap0(χ,Istim) for $I_{\text{stim}}$Istim outside the experimental range $\left[ I_{\text{stim}}^{\text{min}},I_{\text{stim}}^{\text{max}}\right]$Istimmin,Istimmax in the case $a<0$a<0 (PDF 155 KB) [file 11538_2023_1206_MOESM9_ESM.pdf]

Suppl. Section 2: $a < 0$

In this section we carry out the analysis of the Monod function $I^0_{\text{adap}}(\chi, I_{\text{stim}})$ for I_{stim} outside the experimental range $\left[I^{\min}_{stim}, I^{\max}_{stim}\right]$ in the case $a < 0$. Due to the positivity constraint (35) on the Monod function, we consider here only $c > 0$ (otherwise, being the Monod function monotonically decreasing w.r.t. χ as shown in Prop.1, we would immediately have $I^0_{\text{adap}}(\chi, I_{\text{stim}}) < 0$ for all $\chi > 0$).

(2.1) $b > 0$. According to Prop. 1, under these conditions we have that $I^0_{\text{adap}}(\chi, I_{\text{stim}})$ is monotonically decreasing w.r.t. both χ and I_{stim}. The plateau function $P(I_{\text{stim}})$ is also monotonically decreasing. We recall that $\forall I_{stim} \in \left[I^{\min}_{stim}, I^{\max}_{stim}\right]$ and $\forall \chi \in \left[t^{first}_{spk}\left(I_{stim}\right), t^{last-1}_{spk}\left(I_{stim}\right)\right]$ we have that

$$P(I_{stim}) < F(\chi, I_{stim}) < c \text{ and } F(\chi, I_{stim}) < H(I_{stim}).$$

We observe that, with $c > 0$ and $I_{\text{stim}} > I^{\max}_{\text{stim}}$, the Monod function remains positive only if the plateau function remains positive, i.e. when

$$a < 0, b > 0, \quad c > -a \quad I_{stim} < \frac{\log\left(-c/a\right)}{b}.$$

If this is not the case, the decreasing plateau might become negative, leading to negative values of the Monod function as well as for $\chi > t^*$ for some $t^* > 0$. In this case, it is possible to shift the Monod function as done in Eq. (48) by defining

$$c^* := \frac{\alpha}{\beta} + \eta I^0_{dep} + \frac{\delta}{\beta}\left(1 + V^0\right), \quad \eta \in [0, 1], \tag{62}$$

as long as the positivity of the Monod function is preserved; with this choice, both Eqs. (35) and (37) hold. Alternatively, it is possible to ensure at least the positivity of the Monod function by modifying it in a way that the plateau is shifted to zero, i.e. by choosing c^* in Eq. (48) such that

$$c^* = -a \exp(b I_{stim}) > 0. \tag{63}$$

As for $I_{\text{stim}} < I^{\min}_{\text{stim}}$, the plateau remains positive as long as $P(I^{\min}_{\text{stim}}) > 0$, so the only condition to verify is Eq. (37). If Eq. (37) does not hold, it is possible to modify the Monod function by applying the shift (48) with c^* as in Eq. (63).

(2.2) $b < 0$. In this case, according to Prop. 1, we have that $I^0_{\text{adap}}(\chi, I_{\text{stim}})$ is monotonically decreasing w.r.t. χ and increasing w.r.t. I_{stim}. Moreover, the plateau function $P(I_{\text{stim}})$ is also monotonically increasing. In this case, the Monod function remains positive for any $I_{\text{stim}} > I^{\max}_{\text{stim}}$ as long as

$I^0_{\mathrm{adap}}(\chi, I^{\mathrm{max}}_{\mathrm{stim}}) > 0$, $\forall \chi > 0$. This last condition is automatically verified when the plateau function associated to $I^{\mathrm{max}}_{\mathrm{stim}}$ is positive, i.e.

$$P\left(I^{\mathrm{max}}_{\mathrm{stim}}\right) > 0 \Longrightarrow 0 < P\left(I^{\mathrm{max}}_{\mathrm{stim}}\right) < P(I_{\mathrm{stim}}) < F(\chi, I_{\mathrm{stim}}) < c.$$

Equation (35) is hence in this case verified. For Eq. (37), it holds automatically if the threshold associated to $I^{\mathrm{max}}_{\mathrm{stim}}$ is positive, i.e.

$$c \leq H\left(I^{\mathrm{max}}_{\mathrm{stim}}\right) \Longrightarrow c \leq H\left(I^{\mathrm{max}}_{\mathrm{stim}}\right) \leq H\left(I_{\mathrm{stim}}\right), \quad \forall I_{\mathrm{stim}} > I^{\mathrm{max}}_{\mathrm{stim}}.$$

In the case of stimulation currents below the experimental range – i.e. $I_{\mathrm{stim}} < I^{\mathrm{min}}_{\mathrm{stim}}$ – the plateau might become negative after a certain instant t^*, violating Eq. (35). Moreover, Eq. (37) might also not hold. In these cases, it is possible to apply the transformation defined in Eqs. (48)-(63).
